# Supplementary figures and images for: Artificial selection reveals complex genetic architecture of shoot branching and its response to nitrate supply in Arabidopsis
Source: PLoS Genet. 2023 Aug 24;19(8):e1010863. doi: 10.1371/journal.pgen.1010863 (PMC10482290; doi:10.1371/journal.pgen.1010863)

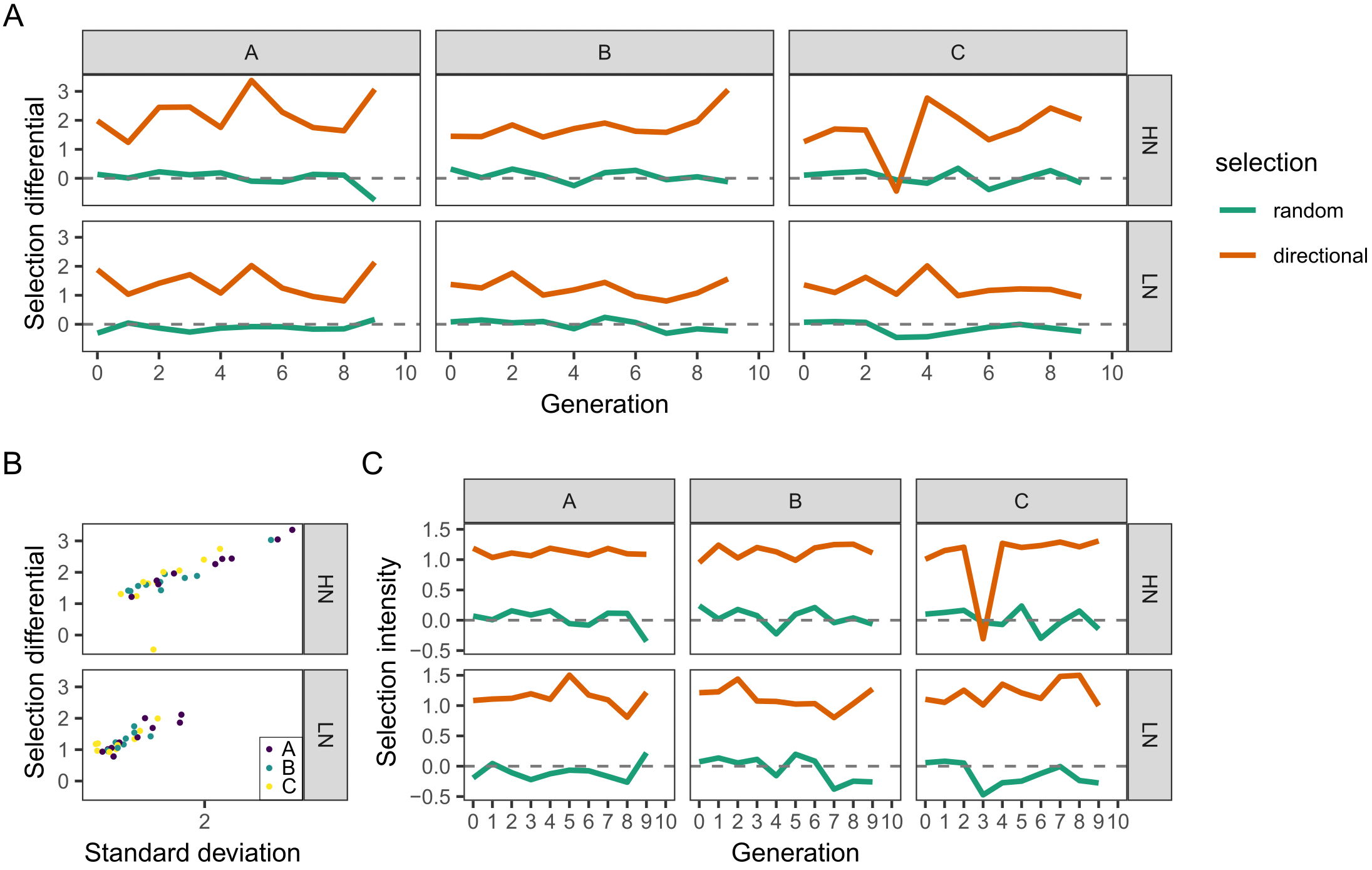

Supplement: S1 Fig — (A) Selection differential across the generations, calculated as the difference between the mean number of branches of selected individuals and the overall mean number of branches of the population in each respective generation. For each replicate, the selection differential is shown for selected and control populations, which for the latter fluctuate around zero as expected, shown by the dashed line. Populations were selected under two different nitrate supply regimes, low (LN) and high (HN). There were independent populations, A-C, for each selection regime and N environment, making a total of six populations. (B) Relationship between variation in shoot branch number (measured as standard deviation) and the imposed selection differential. The selection differential is strongly correlated to trait variation. Coloured points distinguish the 3 replicate populations. (C) The selection intensity (calculated as the selection differential shown in B divided by the trait standard deviation). Selection intensity is relatively constant across the generations and between selection environments. There is an exceptional dip in selection intensity in generation 3 of replicate C on HN, which was due to a randomisation error assigning individuals to crosses (see methods). (TIF) [file pgen.1010863.s001.tif]

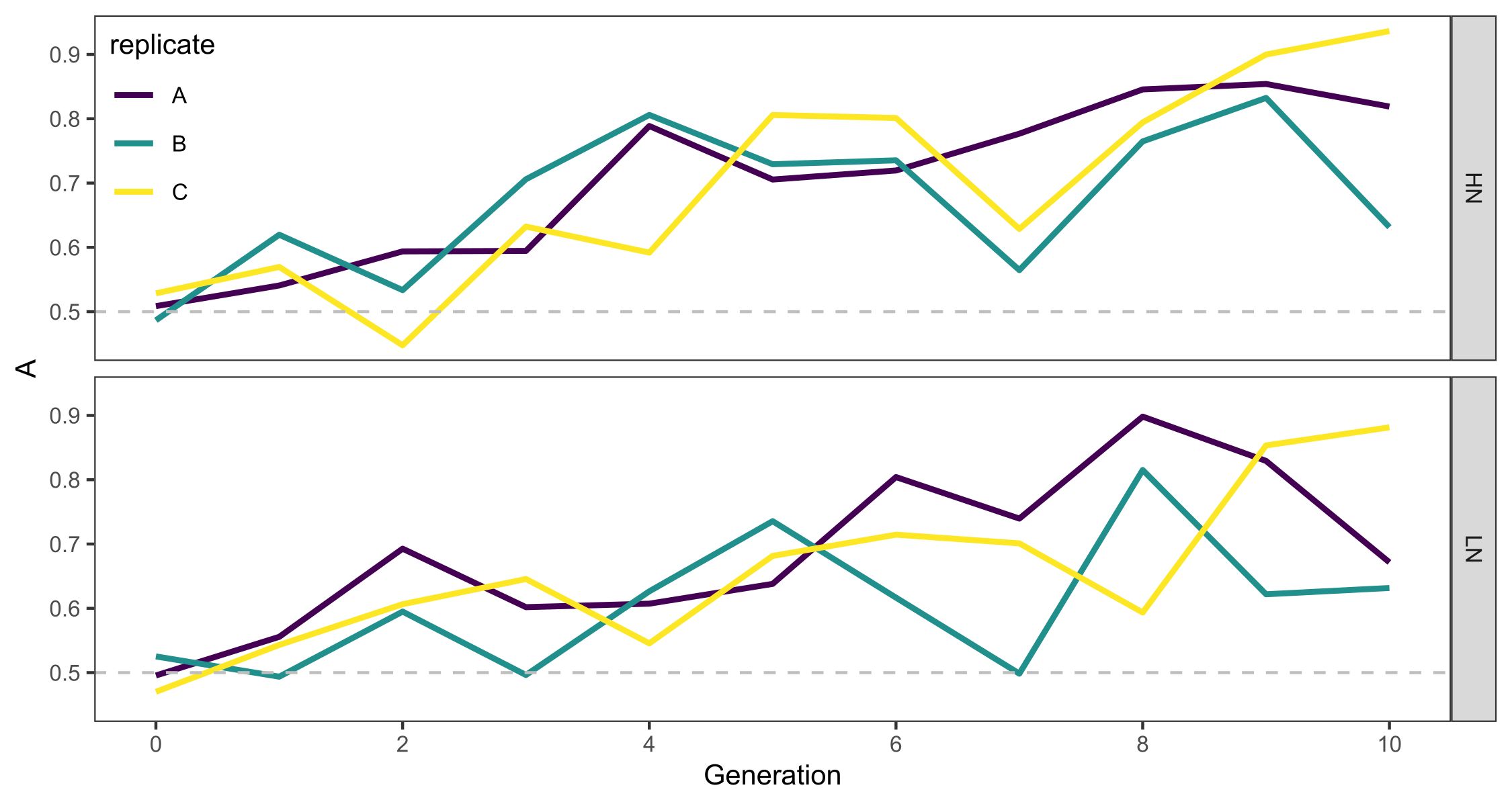

Supplement: S2 Fig — The response to selection measured using the Vargha-Delayney’s A statistic [28], a robust non-parametric effect size measure expressing the probability that an individual from the selected population has a higher number of branches than one from the control population. Replicate populations are coloured as indicated in the key (populations from the same replicate were grown simultaneously in the glasshouse). The grey dashed line represents A = 0.5, which is the null expected value of no effect of selection. (TIF) [file pgen.1010863.s002.tif]

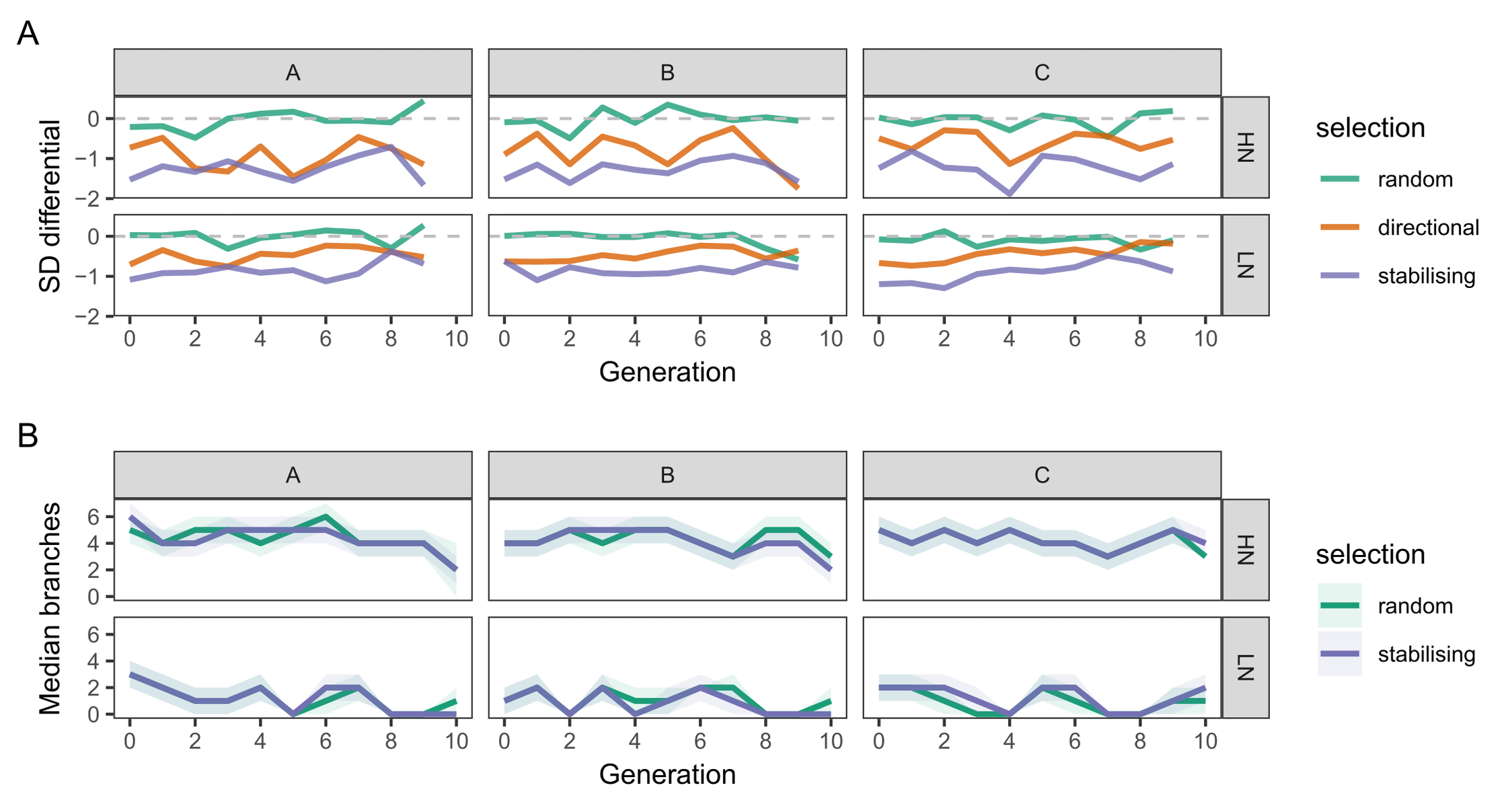

Supplement: S3 Fig — See S1 Appendix for more details about this figure. (A) Standard deviation (SD) differential for shoot branches, calculated as the difference between the standard deviation in shoot branch number for the selected individuals minus that of the overall population. Data are shown for the directional selection populations, for the stabilising selection populations and for the random controls. In both kinds of selection there is a negative SD selection differential, which is as expected: in the case of stabilising selection we sampled from the centre of the distribution; under directional selection, we sampled from the upper tail of the distribution. In both cases, there is an imposed differential selection on the dispersion of shoot branching. As expected, there was no SD selection differential in control populations. (B) Changes in the median number of branches in the random control populations, and populations selected for average branch numbers across the generations. The shaded areas show the median absolute deviation (a robust dispersion measure analogous to the standard deviation). Stabilising selection had no effect on the median number of branches, which might have been expected if random selection had affected the mean of the trait differently from the imposed stabilising selection. In all panels data are shown for three replicate experiments (A, B, C) and for populations grown on high (HN) and low (LN) nitrate. (TIF) [file pgen.1010863.s003.tif]

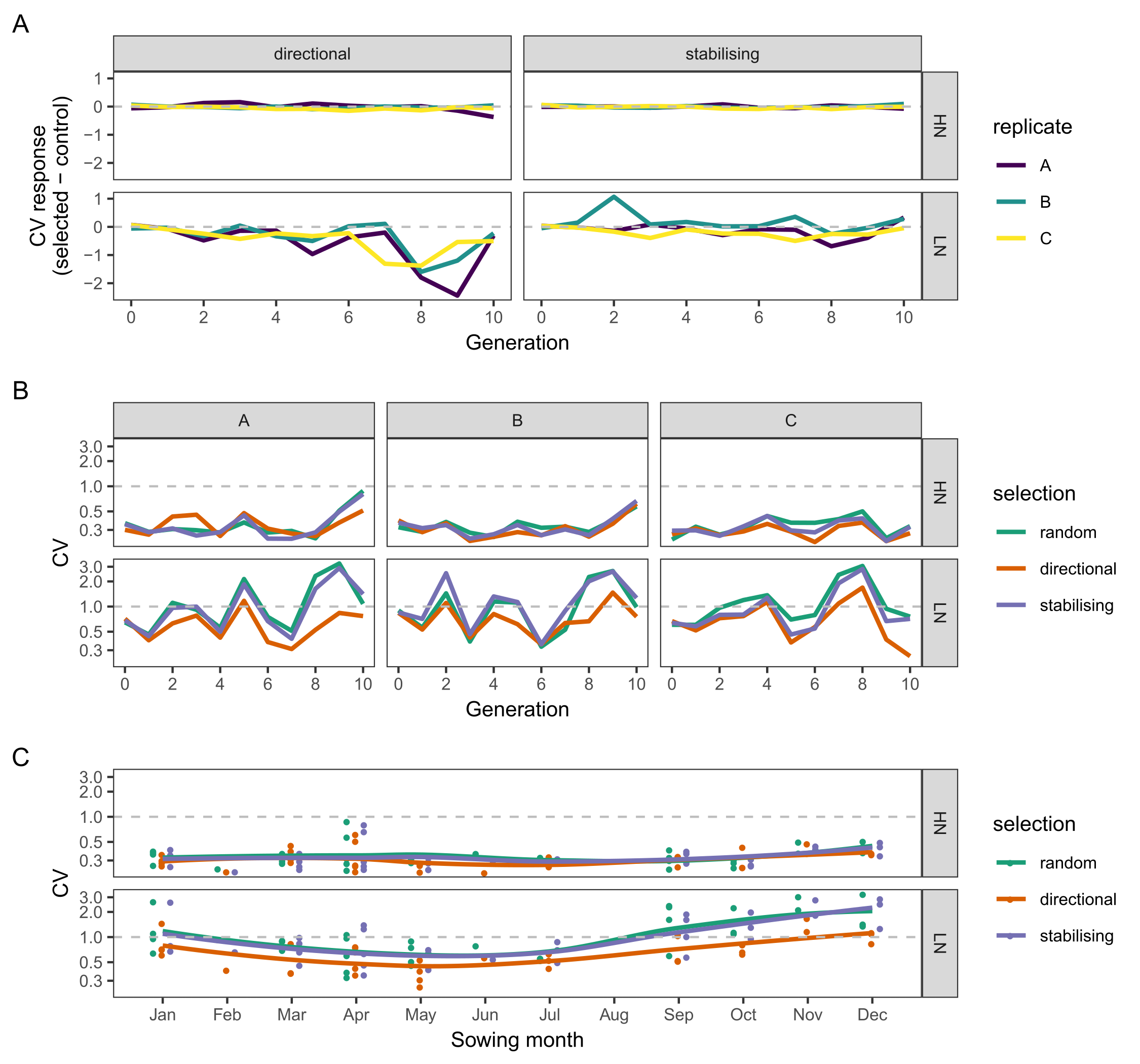

Supplement: S4 Fig — See S1 Appendix for more details about this figure. (A) Effect of selection on shoot branching variability, measured as the coefficient of variation (CV = standard deviation divided by the mean). The response to selection was calculated as the difference in CV between populations subjected to selection (directional or stabilising) and the respective controls in each generation. (B) Coefficient of phenotypic variation in shoot branch number in each population across the generations. (C) Correlation between the sowing month and shoot branching CV. Each point is the CV calculated for one generation and population. The trend lines are a smooth fit by local weighted regression. (TIF) [file pgen.1010863.s004.tif]

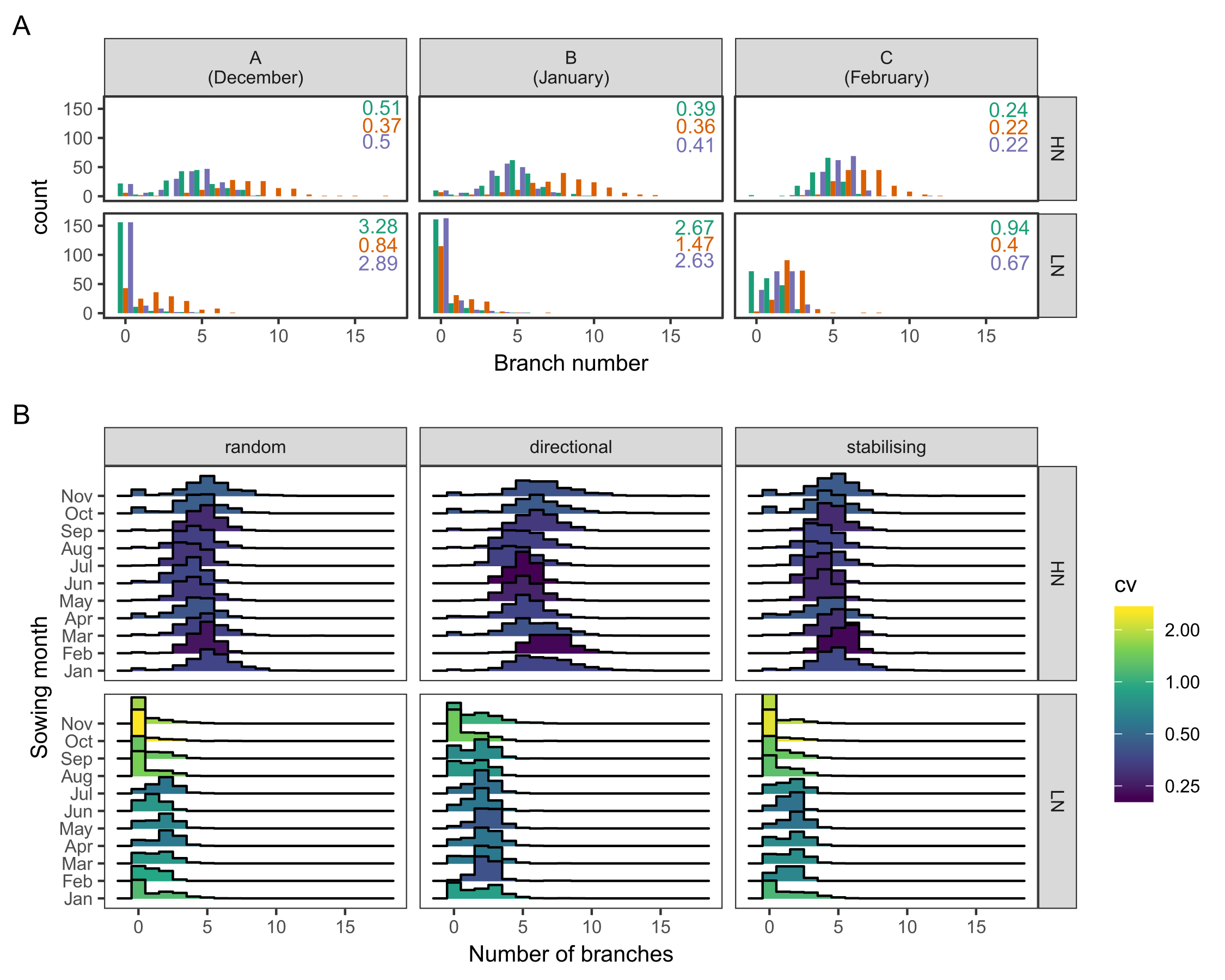

Supplement: S5 Fig — See S1 Appendix for more details about this figure. (A) Examples of distributions of shoot branch numbers from HN and LN populations with different CV values. The distributions shown are for selection generation 9. The numbers within each panel are the CV of the respective distributions. The sowing months are indicated for each replicate. (B) Distribution of shoot branch number by sowing month shown as histograms coloured by the CV. On low N, distributions are highly skewed (leading to a high CV), with a mode of zero branches in the Autumn/Winter months. Populations selected for high branching on low N have a heavier upper tail than the unselected control or those selected for average branch numbers. Replicates and generations were combined in these plots, since we were interested in exploring the marginal effect of scoring season on shoot branching. (TIF) [file pgen.1010863.s005.tif]

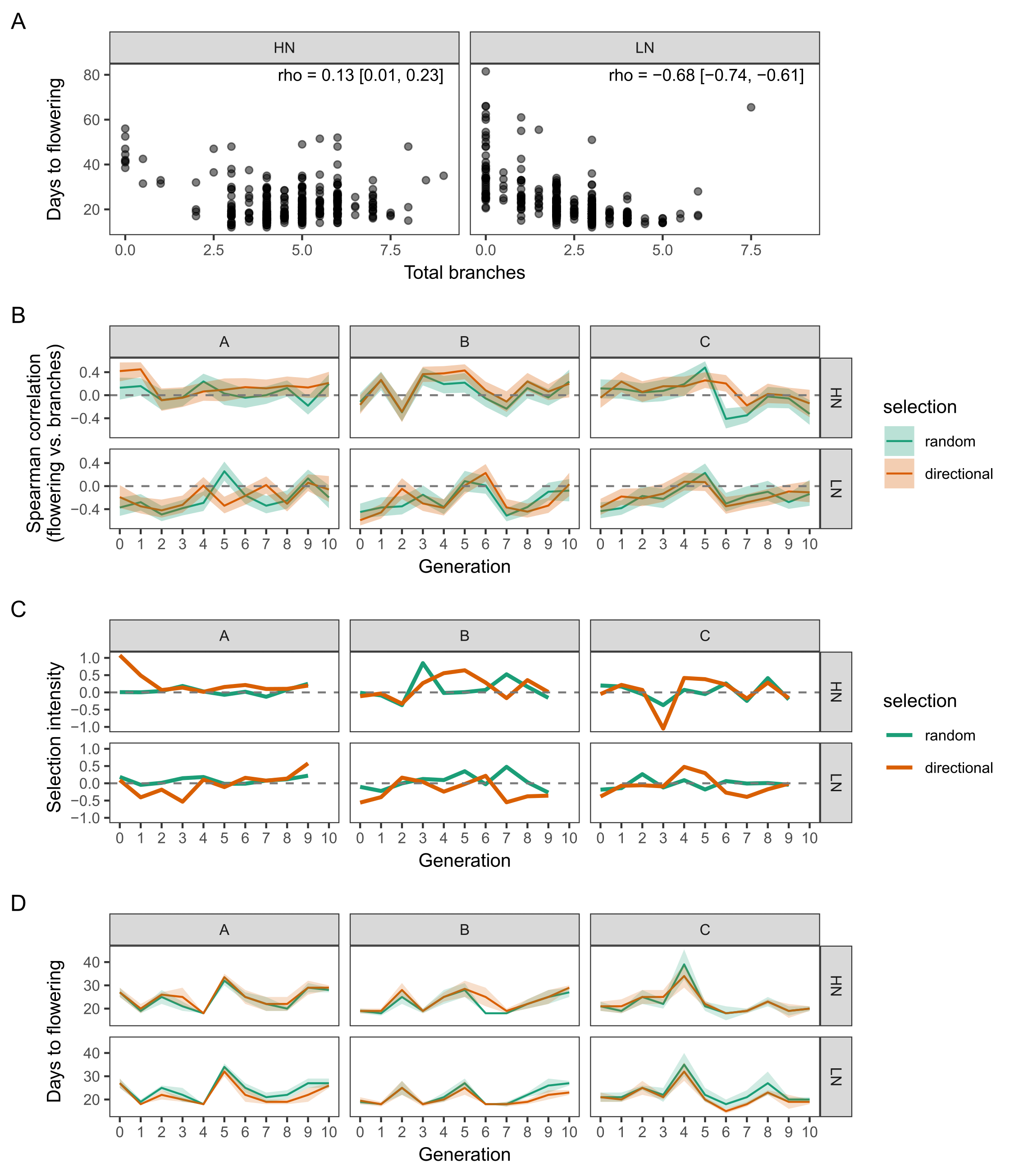

Supplement: S6 Fig — (A) Scatterplot of number of shoot branches versus days from sowing to flowering for 357 of the MAGIC lines that founded the selection populations. Data are the median of each trait calculated from 4–8 replicates of each MAGIC line (median n = 7). The rank-based Spearman’s correlation (rho) is shown in each panel with 95% bootstrap confidence intervals in brackets. Data are from [23]. (B) Spearman rank-order correlation between shoot branching and days to flowering across the generations in the selected populations. The shaded areas show the 95% bootstrap confidence intervals (1000 bootstrap samples). The dashed line at zero indicates no correlation. (C) Selection intensity for days to flowering across the 10 generations. (D) Changes in median days to flowering across the generations for populations selected for increased branching and random control populations. The shaded areas show the median absolute deviation (a robust dispersion measure analogous to the standard deviation). (TIF) [file pgen.1010863.s006.tif]

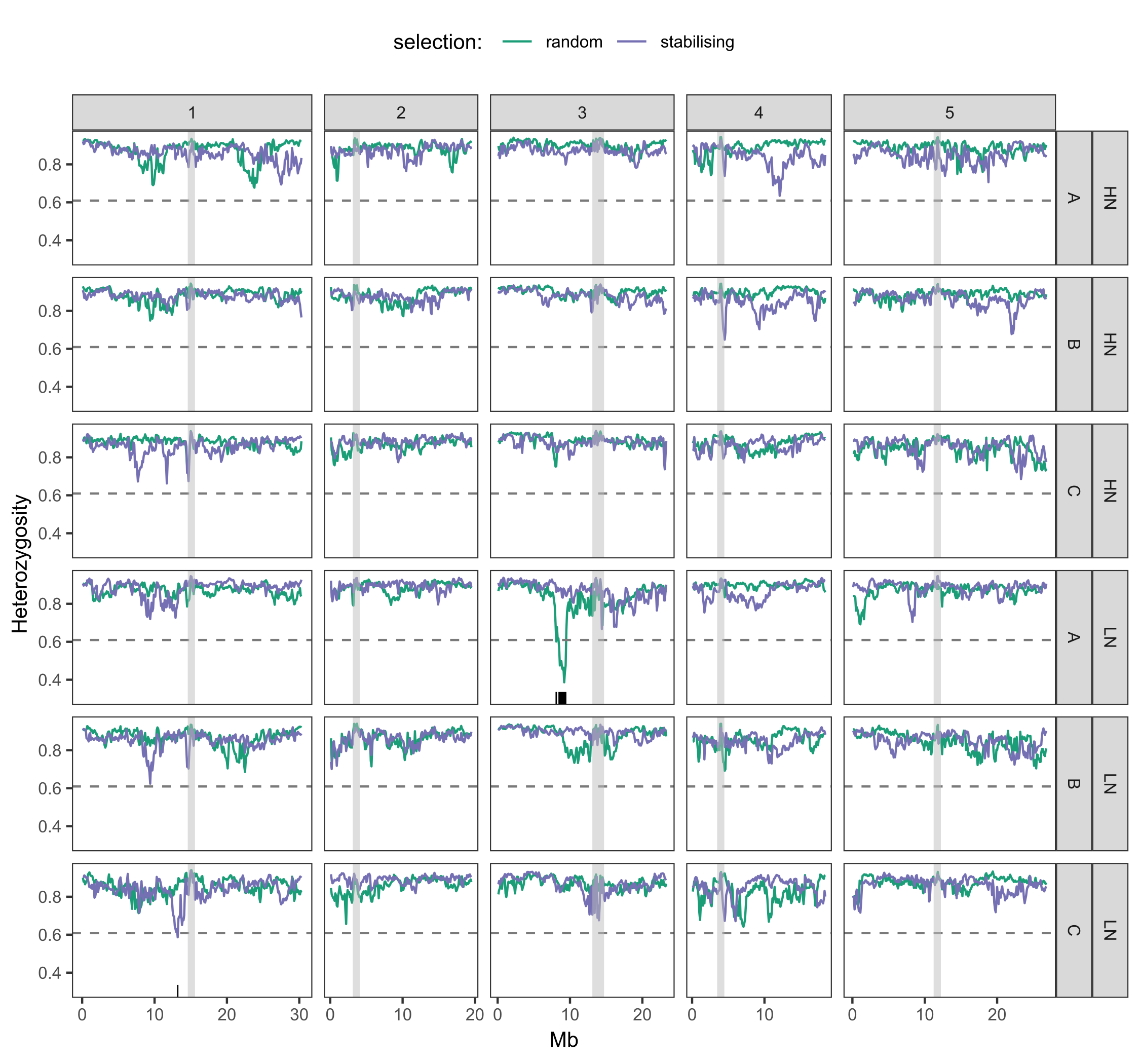

Supplement: S7 Fig — Identical to Fig 4 in the main text, but showing the result for populations under stabilising selection. (TIF) [file pgen.1010863.s007.tif]

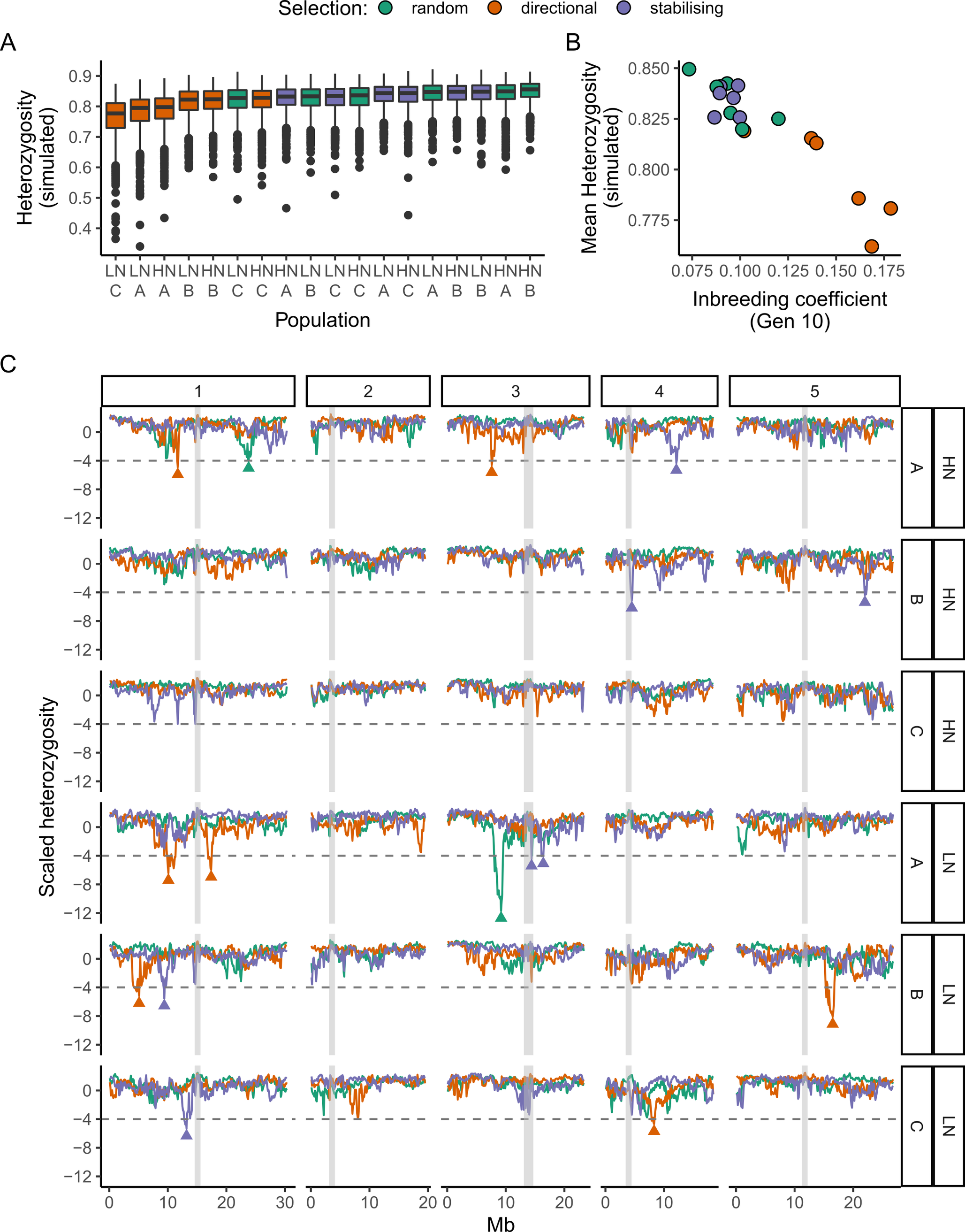

Supplement: S8 Fig — A) Neutral heterozygosity distributions for 1000 simulated loci obtained by using random “inheritance” on each population’s pedigree. Populations are ordered by their median heterozygosity. The ranking is similar to the empirical data shown in Fig 4A. B) Correlation between simulated heterozygosity and inbreeding coefficient of each population, showing that these simulations recapitulate what is observed empirically (compare with Fig 4C). C) Empirical genome scans of heterozygosity for each population, scaled by their respective simulated neutral distributions. The scaled values represent the number of standard deviations the observed heterozygosity is away from the mean of the simulations. The arrowheads highlight the windows falling below -4 standard deviations (dashed line). Data show estimates in 200Kb sliding windows with a 100Kb step. See methods for further details on the simulations. (TIF) [file pgen.1010863.s008.tif]

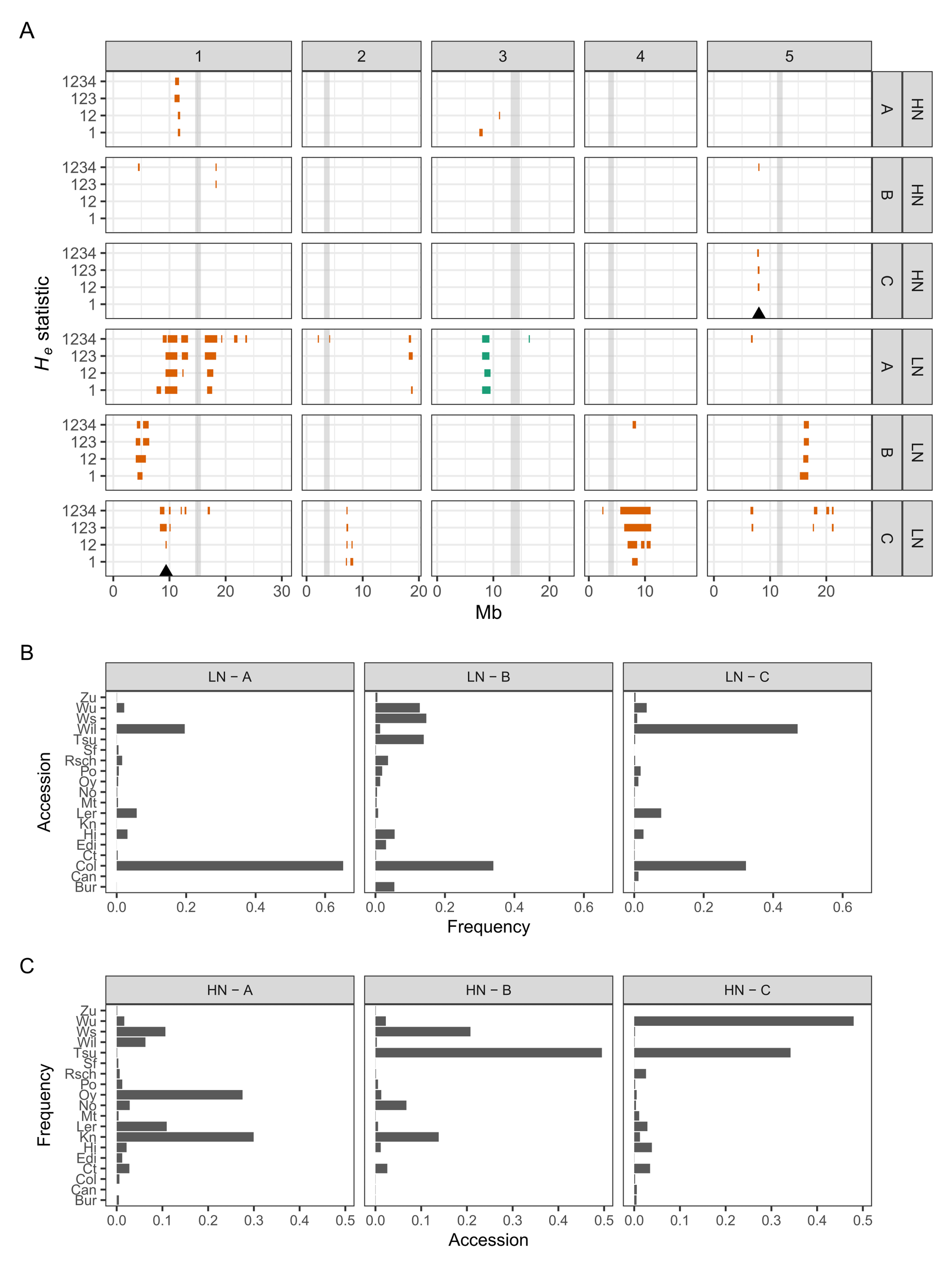

Supplement: S9 Fig — A) Putative selective sweep blocks identified with modified He statistics [35]. These are calculated as previously described, but adding the frequency of the 2, 3 or 4 most common alleles as if they were a single allele (He12, He123 and He1234, respectively along the y-axis). For example, if there was a sweep where 4 alleles were equally selected to the detriment of the other 15 accession alleles, adding their frequencies should create a very common “meta-allele”, resulting in low expected heterozygosity. Similarly to Fig 4 in the main text, the blocks were identified based on falling below 1% of the distribution across selected populations. Blocks within 400Kb of each other were merged together. Shaded grey regions show 1Mb around the annotated centromeric regions. B) Allele frequency spectra at ~10cM on Chr1, which has common sweeps between LN—A and LN—C populations, highlighted with an arrow head in panel A, and corresponding to the region of peak #3 in Fig 5A of the main text. Population LN—B is also shown, as it shows some similarities in its allele frequency spectrum to the other two LN populations. C) Similar to panel B, but for a shared weak signal on Chr5 of HN populations (indicated by an arrow head in panel A). (TIF) [file pgen.1010863.s009.tif]

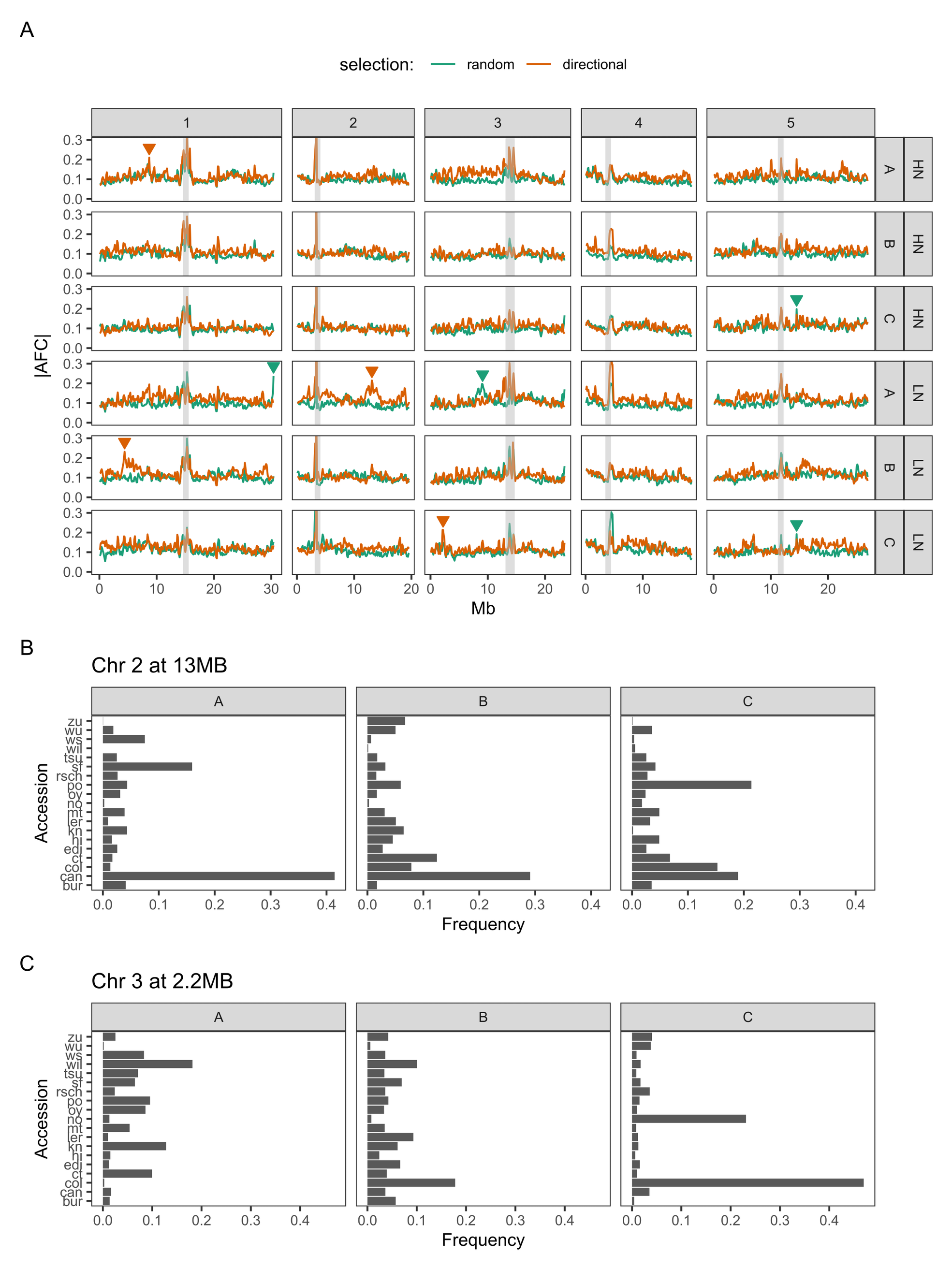

Supplement: S10 Fig — These scans are similar to the heterozygosity-based analysis (Figs 4 and S9), but focusing on biallelic SNP changes (rather than accession-based frequencies). We show the absolute frequency change—|AFC|—between generation 10 (estimated from the Pool-seq data [36]) and generation 1 (inferred based on the SNP allele carried by each accession). A) Mean |AFC| summarised across 200kb windows (step size of 100kb). Outlier windows were defined as those falling above the 99% percentile of the distribution of |AFC| in each selection regime. These are indicated with an arrow head. Some of these peaks had also been identified with the heterozygosity-based analysis, but two are new: on Chr 2 population LN-B; on Chr 3 population LN-C. B) and C) show the estimated frequencies of each accessions’ allele for these two new peaks on all three LN populations, with chromosome and approximate position shown on each plot’s title. (TIF) [file pgen.1010863.s010.tif]

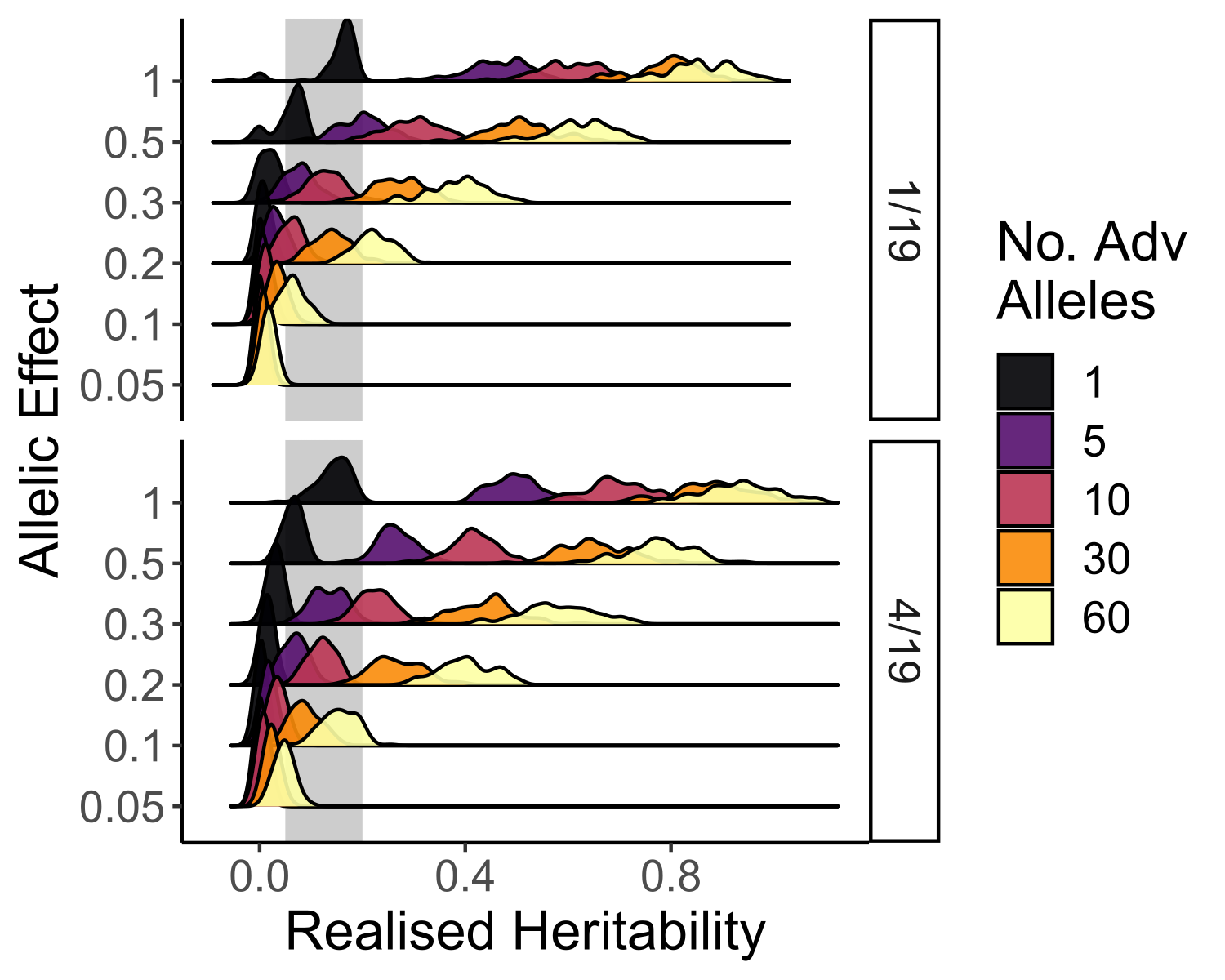

Supplement: S11 Fig — The varying parameters in our simulations were: number of loci affecting the trait (colours); the effect of each of those alleles on the trait (y-axis), which was scaled as the number of standard deviations of increase above the trait’s mean; and the number of accession alleles at a locus that increase the average trait value (panels), which is equivalent to the starting frequency of the advantageous allele in the founder population (all accession alleles start at an equal frequency of 1/19 to capture the random breeding of the MAGIC lines [26]). The grey shaded area highlights the interval [0.05, 0.2], which is broadly compatible with our empirical estimates of realised heritability (Fig 1D). (TIF) [file pgen.1010863.s011.tif]

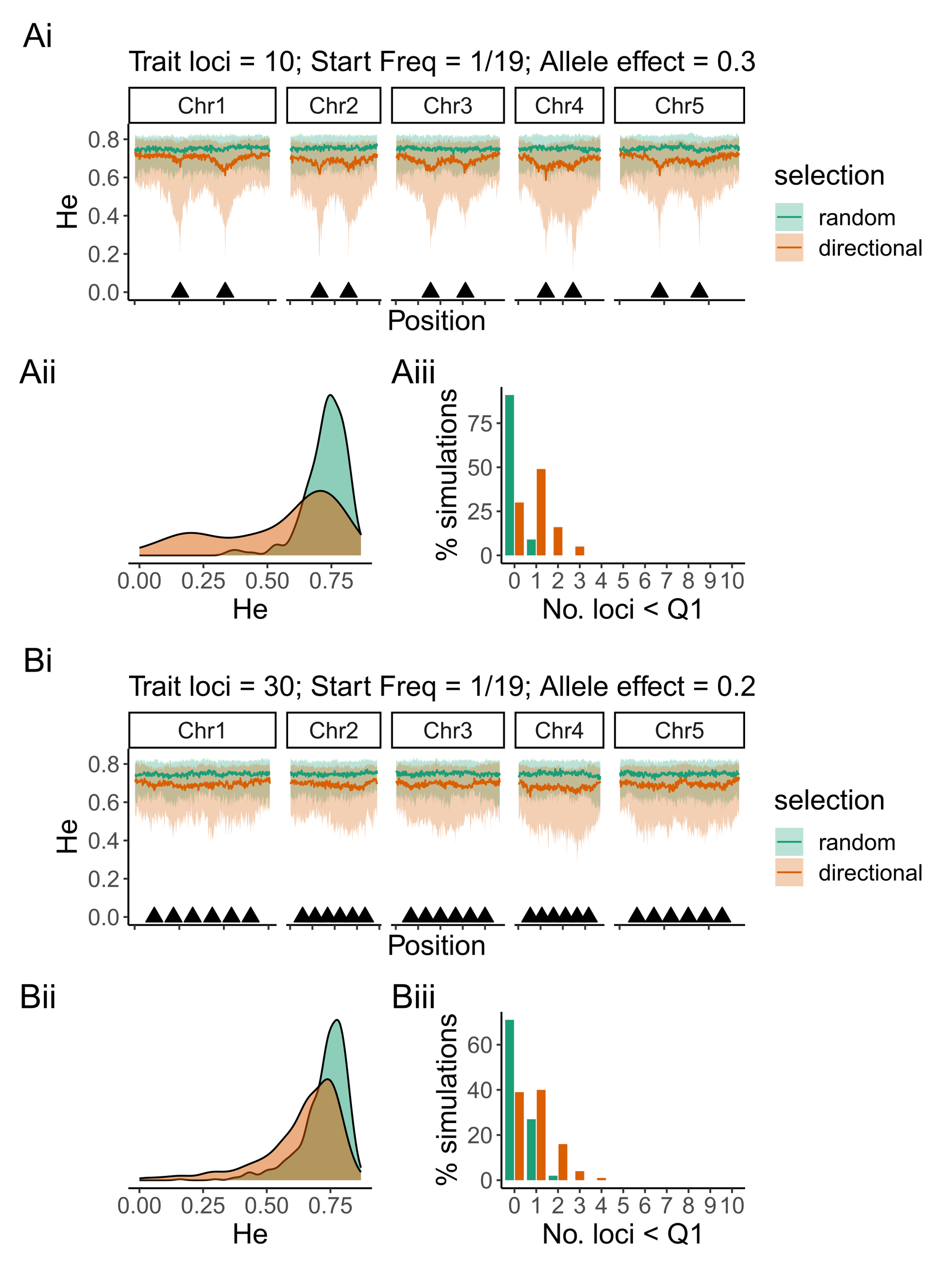

Supplement: S12 Fig — A) and B) show results for different parameter combinations as labelled in the plot titles. Panels i) show average genomic pattern of heterozygosity across 100 replicate simulations under random or directional selection. The line shows the median value and the shaded regions the 10% and 90% percentiles across replicate simulations. The upper triangles show the location of trait loci. Panels ii) show the distribution of heterozygosity at one of the trait loci (the first locus on Chr1, but the distributions are similar for the other trait loci). Panels iii) show the percentage of simulations with a certain number of trait loci falling below the 1-percentile of the genome-wide distribution of heterozygosity. Note that in A) most replicate simulations have at least 1 trait locus below this threshold and heterozygosity distributions are largely non-overlapping. Whereas in B) there is a higher overlap in heterozygosity distributions and in the number of simulations with no trait loci below 1-percentile. (TIF) [file pgen.1010863.s012.tif]

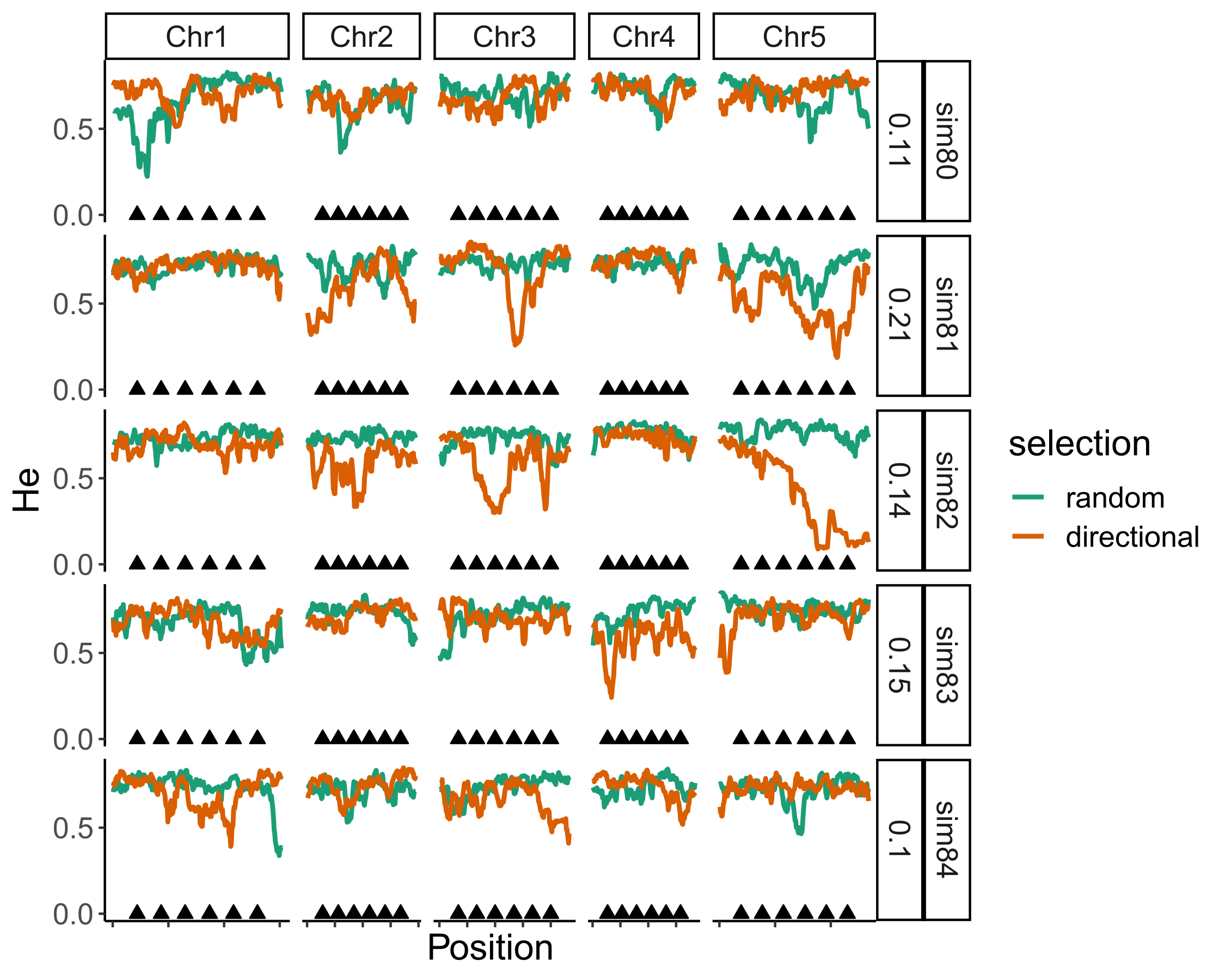

Supplement: S13 Fig — These are individual replicates from the simulation average shown in S12B Fig. Notice how in some of the simulations the signal around trait loci is hard to distinguish from the background (most strikingly for sim82, but to an extent also sim83 and sim84). The signals are also very inconsistent across replicates (i.e. different trait loci are selected for each time). (TIF) [file pgen.1010863.s013.tif]

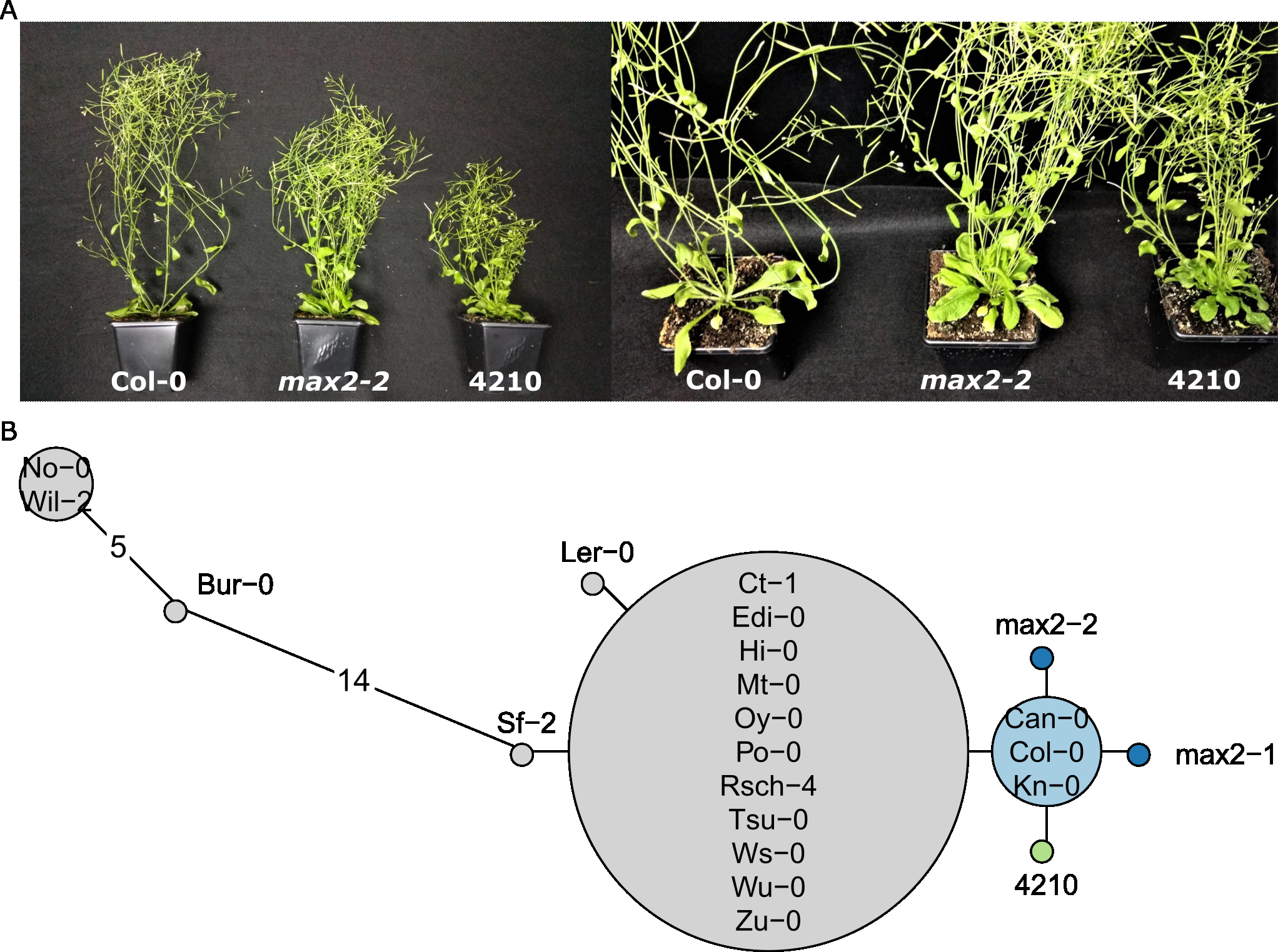

Supplement: S14 Fig — See S2 Appendix for more details about this figure. A) Representative photos of Arabidopsis lines Col-0 (wild-type), max2-2 mutant, and an inbred line (ID 4210) that was produced from single seed descent of individuals carrying a new MAX2 mutation that arose during the selection experiment. Plants were grown on compost for 8 weeks. The photo on the left highlights the increase in branch number and shorter height of the mutants; the photo on the right highlights the difference in leaf shape. B) DNA haplotype network of known MAX2 alleles based on publicly available data. The haplotypes are for the 19 accessions that founded the MAGIC lines. Each node of the network represents a haplotype (size proportional to the number of accession/mutant alleles), and edges connect closest haplotypes (based on the Hamming distance between each allele). The size of the edges is proportional to the number of polymorphisms separating each allele (indicated on the edge, except for alleles with only 1 polymorphic difference). The node containing the standard wild-type accession allele, Col-0, is in blue, with two EMS-derived mutant alleles in dark blue (max2-1 and max2-2). The new allele is shown in green. (TIF) [file pgen.1010863.s014.tif]
