# Supplementary material for: Artificial selection reveals complex genetic architecture of shoot branching and its response to nitrate supply in Arabidopsis
Source: PLoS Genet. 2023 Aug 24;19(8):e1010863. doi: 10.1371/journal.pgen.1010863 (PMC10482290; doi:10.1371/journal.pgen.1010863)
Supplement: S1 Table — See Fig 5A for further details. (PDF) [file pgen.1010863.s015.pdf]

Table S1. Location of candidate selective sweeps in populations under directional selection. See Fig 5A for further details.

Nitrate	Replicate	Chrom	Start	End	Peak ID
LN	B	1	4.3	5.2	1
LN	A	1	7.7	8.5	2
LN	A	1	9.2	11.4	3
HN	A	1	11.5	11.9	4
LN	A	1	16.7	17.6	5
LN	C	2	7.0	7.2	6
LN	C	2	7.8	8.3	7
LN	A	2	18.6	18.9	8
HN	A	3	7.5	8.1	9
LN	C	4	7.7	8.7	10
LN	B	5	15.3	16.8	11
