# Supplementary material for: Artificial selection reveals complex genetic architecture of shoot branching and its response to nitrate supply in Arabidopsis
Source: PLoS Genet. 2023 Aug 24;19(8):e1010863. doi: 10.1371/journal.pgen.1010863 (PMC10482290; doi:10.1371/journal.pgen.1010863)
Supplement: S1 Appendix — (PDF) [file pgen.1010863.s016.pdf]

S1 Appendix. Effects of selection and environment on shoot branching variability

The effects of selection impact not only the trait mean, but also its (genetic) variance, which is expected to decrease over time [1]. Thus, we assessed the impact of selection on the variability of shoot branching, including in populations subjected to stabilising selection (selecting individuals with a number of branches closest to the population's average, S3 Fig). To assess the impact of selection on trait variance independently from mean, we calculated the coefficient of phenotypic variation (CV), by scaling the standard deviation in each population and generation by their respective means. Compared to control populations, only the group of populations under directional selection on LN had a noticeable response in CV, becoming relatively lower across the generations (S4A Fig). Despite the imposed stabilising selection, there was no detectable response in the dispersion of branch numbers in these populations, which were indistinguishable from the control populations (S4A Fig).

In absolute terms, the populations grown on the two nitrate treatments differed in their CV, regardless of the selection regime (S4B Fig). While on HN the standard deviation of shoot branching was always lower than the mean (CV < 1), for populations grown on LN, there were big fluctuations between generations, with standard deviations often greater than the mean (CV > 1). An analysis of the distribution of shoot branching revealed that this is mostly because, on LN, many individuals do not branch, resulting in severely skewed distributions with a mode of zero branches (e.g. S5A Fig, LN panel). Directional selection shifts these distributions away from zero, changing their shape with a resulting lower CV. Under HN conditions, directional selection also shifts trait distribution, but its shape remains similar, having little impact on CV (e.g. S5A Fig,

HN panel). Therefore, by using CV as a measure of phenotypic variation we in fact captured features of the trait distribution, particularly its skew, which is high on LN.

The variation in CV among LN populations was also strikingly cyclical through the generations, regardless of selection regime (S4B Fig). Because successive generations were grown in the glasshouse across a range of seasons, this suggested an environmental influence on shoot branching. Indeed, LN populations grown in Autumn/Winter months had on average higher CVs, and showed more skewed distributions than those grown during Spring/Summer months (S4C and S5B Fig). This relationship between the distribution of branching and the seasonal differences across the generations seemed to affect all LN populations similarly, regardless of the selection regime. Although these experiments were not designed with the purpose of testing for seasonality effects, our data reveal potential interactions between shoot branching and multiple environmental variables, here represented by the nutrient treatment and seasonal conditions.

The skewed distributions with a mode of zero on LN may explain why the effect of selection for increased branching described above was subtle when using the median of the population, (which is often close to zero in all populations). In these cases, the effect of selection is clearer by considering that the tails of the selected populations are longer than those of the random populations (e.g. lower left panel in S5A Fig), which the robust non-parametric effect size measure we used captured well (S2 Fig).

References

1. Barton NH, Keightley PD. Understanding quantitative genetic variation. Nat Rev Genet. 2002;3: 11–21. doi:10.1038/nrg700
