# Supplementary material for: Artificial selection reveals complex genetic architecture of shoot branching and its response to nitrate supply in Arabidopsis
Source: PLoS Genet. 2023 Aug 24;19(8):e1010863. doi: 10.1371/journal.pgen.1010863 (PMC10482290; doi:10.1371/journal.pgen.1010863)
Supplement: S2 Appendix — (PDF) [file pgen.1010863.s017.pdf]

S2 Appendix. A new shoot branching mutant did not affect selection signals

In our analysis, we ignored the role of new mutations in the selection process, given the relatively small effective population size and short number of generations. However, one new mutation arose in population LN-C under directional selection, made evident by an unusual phenotype detected in a family of individuals in the 10th and final generation. These plants were shorter, with rounded leaves and increased branching compared to other individuals from the same population (S14A Fig). Four siblings in this generation had a median of ~10 branches, while the median for the rest of the population was only ~3 branches. This phenotype resembled that of known mutants impaired in the perception/signalling of the strigolactone hormone. To confirm the occurrence of a new mutant in one of these strigolactone-related genes, we performed test-crosses to mutant lines of *max1-1*, *max2-1*, *max3-9*, *max4-5*, *d14-1* as well as the standard lab accession Col-0 as a control. All complemented the new candidate mutant, which is recessive, apart from *max2-1*, suggesting a new mutant allele in this gene.

To exclude that this was due to seed contamination in our population, we Sanger-sequenced a genomic fragment covering the *MAX2* locus of lines carrying the new allele. Indeed, we found a novel mutation, distinct from the known *max2-1* (Asp-581-Asn) and *max2-2* (Trp-585-Stop) mutant alleles. This mutation induces a change from Glycine to Arginine at amino acid 369. We were able to track the mutation back to at least generation 4, by genotyping individuals re-grown from seed stock of crosses from earlier generations. Comparing its sequence with that of the 19 founder accessions of our population, the new allele derives from a *MAX2* haplotype shared by Can-0, Col-0 and Kn-0 accessions (S14B Fig). Based on diagnostic SNPs in the neighbouring

region of the gene, we were able to confirm that the background of the mutation was Col-0.

We made a rough estimate of the chances of obtaining such an extreme mutation in our experiment. Assuming a mutation rate in Arabidopsis of around 7e-9 per site per generation [1], with 6 populations selected for increased branching, each of 200 individuals (400 gametes), selected over 10 generations, then the chance of a mutation occurring in a specific site across the whole experiment is ~0.02%. We can multiply this number by the number of mutations likely to result in extreme branching phenotypes as observed. For example, there are ~1500 possible mutations resulting in a premature stop codon in genes known to be involved in shoot branching (*MAX1*, *MAX2*, *MAX3*, *MAX4*, *D14* and *BRC1*). This results in a ~30% chance of a mutant allele in one of those genes. Despite the simplifications of this calculation (e.g. assuming that any premature stop codon would result in a null allele, and ignoring other mutations that may equally disrupt these proteins' functions), this shows that an extreme mutation might be picked up in such a large experiment.

However, it is worth noting that any new mutation would have to overcome the effects of drift and, for recessive mutations, reach homozygosity to contribute to selection for the trait of interest, lowering its effective chance of being fixed in the population. Indeed, the extreme branching phenotype of these individuals was only detected in the last generation of our experiment. Also, there was no clear selection signal in the location of this gene in the respective population (Chr2 ~17.7Mb, cf. Fig 4). Therefore, although new mutations may arise in the population, they are unlikely to be the main drivers of the genomic signals we described, and may have only contributed to trait

changes if the experiment had continued for further generations, increasing the probability of their fixation.

References

1. Ossowski S, Schneeberger K, Lucas-Lledó JI, Warthmann N, Clark RM, Shaw RG, et al. The rate and molecular spectrum of spontaneous mutations in Arabidopsis thaliana. Science. 2010;327: 92–94. doi:10.1126/science.1180677
