# Supplementary material for: Artificial selection reveals complex genetic architecture of shoot branching and its response to nitrate supply in Arabidopsis
Source: PLoS Genet. 2023 Aug 24;19(8):e1010863. doi: 10.1371/journal.pgen.1010863 (PMC10482290; doi:10.1371/journal.pgen.1010863)
Supplement: Attachment — Submitted filename: 2023-04-15_response_to_reviewers.pdf [file pgen.1010863.s018.pdf]

Dear Magnus and Claudia,

We are pleased to submit a revised version of our manuscript "*Artificial selection reveals complex genetic architecture of shoot branching and its response to nitrate supply in Arabidopsis*" for publication in PLOS Genetics.

We are grateful for the valuable comments that both you and the four reviewers have provided about our manuscript, and for your willingness to consider a revised version for publication in PLOS Genetics. We have made major revisions to the manuscript in response to the comments and feedback given. We hope we have successfully addressed the concerns raised and that our revised manuscript will be suitable for publication.

With best wishes

Ottoline

# Editor's Comments

What needs work is the analysis and interpretation. The paper is currently framed in terms of the unfortunate "missing heritability" debate. I say "unfortunate", because even the people who helped coin this phrase have long since admitted that this simply reflects ignorance of basic statistics and quantitative genetics: you just don't have the power to pick up anything but the largest effects. There is no reason for researchers outside human genetics to be drawn into this discourse. Especially not when you have a design like yours, where power is extremely limited and linkage disequilibrium extensive — a very different setting from enormous human GWAS studies.

We have revised our text to avoid framing this issue around the "missing heritability" idea. We removed one mention to this idea from the introduction, which now says (L62, new text in bold):

"However, the complex genetic architecture of many traits poses challenges in terms of the statistical power to detect such QTL (Yang et al. 2010; Young 2019; Visscher et al. 2017). The genetic control of a trait may involve many loci, each having a small effect, with some alleles being rare in the mapping populations. In addition, allelic diversity at each locus, population structure, and the effect of environment on the traits under study all complicate the analysis [5–9]. **However, well-powered analysis, with ever-increasing sample sizes has been fruitful at uncovering the genetics of even highly polygenic traits [10]. Height in humans is an excellent example, where current sample sizes in the millions have uncovered thousands of loci explaining a large fraction of the heritability in this trait [11].**"

We have also framed our discussion of these issues around statistical power limitations, which as the editor and reviewers mention, is the main thing affecting studies like ours.

You keep talking about the architecture being "polygenic", but what do you actually mean by that? Can you put bounds on how many loci are involved, and can you estimate their effect sizes? The latest human height GWAS identified 12k putatively causative loci — perhaps there are 12k loci affecting roots, but you obviously cannot say anything about this. My point here is that "polygenic" ends up being yet-another buzzword, and it would be more helpful to use your simulation results to try to delimit what could actually be going on (to the extent this is possible, and to the extent you want to make claims about it).

We thank the editor for raising this point. We think it is valuable to try to make (some) claims about the genetic architecture of our trait, in particular given the inconsistent signal across replicates in LN populations and the lack of clear sweeps in HN populations. We have followed the advice given and have made our description and discussion of results more focused on what we mean by “polygenic”.

Our simulations now just focus on showing that our data on low nitrate are compatible with a trait controlled by 10-20 loci of equal effect spread across the genome. For high nitrate populations, the picture is complicated by the fact that we had difficulty finding any signal at all (despite similar heritability estimates and selection responses). However, our simulations helped in showing that at around 30 loci, the signals start to be very inconsistent and sometimes even indistinguishable from the background. Based on this, we suggest that the genetic architecture for shoot branching is different depending on the nitrate environment, and discuss this result in light of the possibility that there is conditional neutrality for the loci controlling this trait. This would explain the high gene-by-environment interaction described in previous studies.
In light of this, we have revised Fig S9-S11 to focus more on how the number of loci affects the genomic signals in simulations. In particular S10 now shows simulations that contrast the signals between 10 and 30 loci (whereas the previous version showed 3 and 10 loci). This made more sense, since we detect at least 8 broad peaks in LN, so we know the trait involves at least that many loci. We now include simulations with 30 loci, to show how the inconsistent signal across replicates is exacerbated and sometimes even indistinguishable from the background. We use these simulations to discuss the difference in genomic signals between LN and HN populations.
And we have refrained from making comments about “allelic heterogeneity” in these simulations, which we agree was not properly accounted for or addressed (we briefly discuss the issue of allelic heterogeneity in the discussion).

Likewise you keep mentioning allelic heterogeneity, but what does this actually mean? How large are the haplotypes that are actually segregating in this population, and that are being selected? It seems to me that unless the number of causative sites/loci is very small, you will almost certainly have heterogeneity among haplotypes.

Continuing from the previous comment, we further simplified the manuscript and refrain from making claims about allelic heterogeneity. Indeed, we have made no analysis to address how long the haplotypes are and this is not the main focus of the paper. We bring this possibility into the discussion, but do not develop a strong narrative around it as we did not do any experiments/analysis to back it up.

In general, there is a tendency to try to apply various concepts from population genetics where they do not quite make sense. For example, as noted by Reviewer 1, using heterozygosity to look for "selective sweeps" is odd given that you will have no power to detect anything except the rise of singleton haplotypes.

We appreciate the thoughts expressed by reviewer #2 about this issue. We have justified our choice in the reply to their comments, and incorporated further analysis using a more standard SNP-based allele frequency change approach, often used in the field.

Furthermore, you discuss the possibility of genetic drift without mentioning the classical result that heterozygosity should start at 1 - 1/19, and decrease by a factor (1 - 1/80)^10 — which it doesn't appear to do, suggesting that maybe there is some associative overdominance in the population.

We thank the editor for noting this. We have added the expected value of heterozygosity at generation 10 to Fig 3 and now write (L332):

“The decay in heterozygosity in random populations was less than expected theoretically (lower dashed line): $H_t = H_0(1 - 1/2N)^t$ predicts that $H_{Gen10}$ ~ 0.83 (Gillespie 1998). This may be due to associative overdominance, whereby recessive deleterious mutations are masked by remaining in a heterozygous state, leading to increased heterozygosity in linked regions of the genome (Pamilo and Pálsson 1998).”

It is obviously none of my business to tell you how to interpret your results, as long as all conclusions are backed up by data, but the whole thing feels overwrought, and risks being misleading. Far better to focus the analyses on what conclusions may actually be drawn from the (impressive) data using a simpler and more bottom-up way, then put it into context in the Discussion.

We have made major revisions to our manuscript, in light of this and similar comments from the reviewers. We hope that the new revisions make the manuscript easier to read and mitigate this concern from the editor.

We have highlighted some of the major changes in the re-submitted manuscript and detail them below in response to each of the specific reviewer’s comments.

One major revision we made in order to shorten the main text and streamline its narrative was to move two subsections to supplementary appendix:

- S1 Appendix: “Effects of selection and environment on shoot branching variability”
- S2 Appendix: “A new shoot branching mutant did not affect selection signals”

Both of these included discoveries that were incidental to the main point of the manuscript. Therefore, we thought moving them to supplementary helped strike a balance between including a detailed description of our data while streamlining the flow of the main text. We still refer to the main points from both of these sections in the main text (L215 and L727).

# Reviewers’ Comments

## Reviewer #1

Tavares et al described an artificial selection approach coupled with Pool-seq aiming to identify the genetic components contributing to shoot branching under various nitrogen conditions. The application of artificial selection in plants is rather novel and seems to be a heroic effort. The discovery of a new MAX allele during the artificial selection is interesting.

My major concern of the manuscript is that although the approach is novel, the uncovered genetic insights underlying either shoot ranching or nitrogen response seem to be limited. The independent replicates do not confirm each other to prioritize genomic regions of interest, and the discussion of underlying gene loci in the sweeps was rather limited. This could be caused by minor contribution of many loci, however, it also could be caused by the limitation of experimental system such as the small population size and short number of generations.

The fact that the genomic signals to artificial selection in the different replicates don’t agree with each other is in itself an interesting result, notwithstanding the limited power of the experiment. It shows that allelic combinations and many loci can result in similar branching phenotypes. This is similar to what has been reported in other systems, e.g. in mice (Castro et al 2019) and drosophila (Barghi et al 2019). In fact, Castro et al. say: “The empirical evidence for adaptive genetic redundancy is extremely sparse, but this probably reflects a bias toward methodologies that search for convergent genetic changes”. In that context, we hope that our study contributes to this literature, by providing another example where similar response to selection is underpinned by different genetic makeups in the final populations.

The reviewer also alludes to concerns about the statistical power in our system given the number of generations and population size (an aspect also mentioned by the editor). We agree that this is certainly an issue, and we expanded our discussion on this limitation, with references to the simulation works of Langmüller & Schlötterer (2020) and Barghi & Schlötterer (2020) (L676).

Overall the manuscript read a bit descriptive and technical, which could benefit from editing and streamlining to focus on the central biological question (branching and nitrogen)

In light of this comment and similar comments from the other reviewers/editor, we have revised the manuscript to 1) shorten the results section by moving incidental discoveries to supplementary appendix and 2) shorten and focus the discussion around the main points on the response to selection in the two nitrate treatments.
Please see our reply to the last comment from the editor, for more details on these changes.

About the nitrogen effect, since at least part of the motivation was to study the underlying mechanism of shoot branching plasticity in response to N changes, I was wondering what difference it would make if the selection was done by picking the highly N-responsive plants (ranked by difference between the branching in low N and high N)? At least the author should discuss the logic of the selection design.

It is hard to select for plasticity (i.e. N-responsive plants) as the reviewer suggests, since a given individual can only grow in one of the environments. Measuring individual-level plasticity is only possible with inbred lines, but a selection experiment explicitly relies on breeding recombining populations. Instead, to assess plasticity we measured family-level N-response, by comparing the average branching of full siblings in high and low N at generation 10 (with the reasoning that siblings will be, on average, more genetically similar to each other). We observed a change in this family-level estimate of plasticity in the groups selected on high N, showing high branching on high N is associated with increased N-sensitivity (even though plasticity itself was not the target of selection).
To help clarify this, we have added (L241): "Our reason for estimating family-level plasticity this way is that measuring individual-level plasticity is only possible with inbred lines."

Minor:

1) Authors should describe low N and high N conditions in result session 1

We have now added (L140): "These treatments were applied by a weekly feed containing 9mM or 1.8 mM $NO_3^-$, respectively (see methods)."

2) Figure 6 was chopped off

This has now been fixed.

## Reviewer #2

Tavares et al present results from an artificial selection experiment on shoot branching in Arabidopsis. They monitor 18 populations, characterizing the phenotypic responses to selection and then applying pooled sequencing to the generation 10 descendants. At the trait level, responses to selection were not large, but owing to the high level of power in their design, the authors were able to establish a genetic basis to variation (realized heritabilities > 0.0). This is not the sort of thing that PLoS genetics typically publishes, but I appreciated Tavares et al's thorough workup on the direct and correlated responses to selection.

The genome sequencing of the evolved populations is the second part of the study. Here, I am confused by their approach. Tavares et al focus on He, the amount of haplotype variation within each part of the genome. It is true that classic selective sweeps will cause localized reductions in He. However, this seems a very indirect way to identify selected loci in an experiment that was maintained for 10 generations. What researchers typically look for in these kinds of studies is whether particular alleles change in the same direction within treatments but not across treatments. The 'high allele' for branching at a QTL should consistently increase in the up-selected populations but not control or stabilizing treatments. If 4 of the original 19 lines carried the high allele, the selection populations might show a change from 20% to 50% for these haplotypes (collectively). If this happened consistently across selection replicates, the locus would likely be highly significant by any of the standard evolve-and-resequence tests (reviewed recently by Schlötterer and colleagues), but would show minimal signal in He. These tests can be applied SNP to SNP or on haplotypes. Given

the fairly weak inferences obtained from He, I could not see any reason why these more standard analyses would not work with this dataset.

We thank the reviewer for their thoughtful and questioning comments. We will try to unpack and address the several points alluded to.

Starting with our choice of using He as a metric to present our results. The reason we chose He as a metric in our genome scans was because it allows us to summarise the allele frequencies across 19 alleles as a single number. As He is a fairly familiar metric in the field, we thought this would be a suitable way to present our results. We note several things that led to our choice:

- Since we know the starting population well (MAGIC lines), it made sense to us to focus the analysis on the 19 accession alleles that are segregating in this population, rather than on bi-allelic SNPs.
- In our founding MAGIC RIL population each accession allele is effectively at ~1/19 frequency. Therefore, reporting our analysis as a difference in allele frequency between the start and end would effectively be subtracting a constant (1/19) from our final generation frequency estimate.
- Even if we proceeded that way, we would end up with 19 allele frequency changes, which is hard to report and visualise (as compared to a biallelic locus). Thus our choice to summarise the allele frequencies with a single number, He in this case.
- We could have chosen to report, for example, the maximum allele frequency out of the 19 accession alleles (with the argument that any accession allele selected for would show maximum frequency out of all 19 alleles). However, as expected, this strongly correlates with He (see plot below).
- The statistics used in some of the more recent work of Schotterer and colleagues focuses on biallelic loci, using time series sampling to look at trends in allele frequency change. This is indeed statistically more powerful, but unfortunately we do not have time series sequence data, so cannot apply the same type of methods.

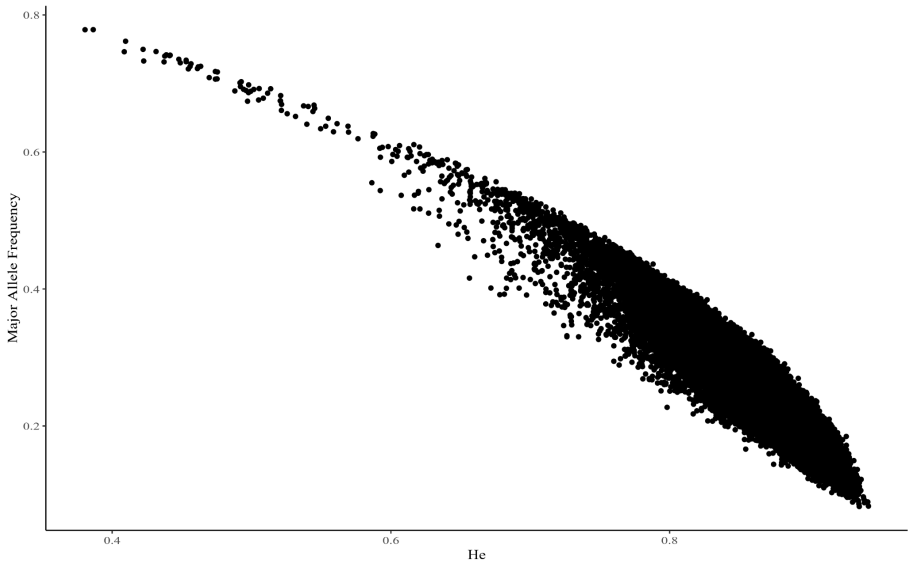


The reviewer also alludes to the fact that we might miss significant signals using He, if several of the accession alleles collectively increase in frequency. This is effectively what we

looked for by using the modified He statistics, where the frequency of the top 2, 3 or 4 most frequent alleles is added up and used to calculate a new He statistic as if they were a “meta allele”. As we’ve shown, this did not reveal a substantially higher number of significant loci (it is generally more difficult to distinguish signal from background).

However, in response to the reviewer’s comments, we have complemented our accession-based analysis with a SNP-based analysis as well. Some points about this analysis:

- We estimated SNP allele frequencies from the Pool-seq and calculated the absolute allele frequency change |AFC| relative to the starting population (similar to Burny et al 2022). In this case, the starting allele frequencies depend on which accessions carry each SNP allele: although ~50% of the SNPs are private to an accession, others are shared by at least two accessions.
- Summarising average |AFC| across sliding windows has its limitations, as most SNPs start at a low frequency and thus are lost by drift (an issue mentioned, e.g. in Burny et al. 2020). E.g., if selection was acting on a SNP starting at 5%, it is still likely that it would be lost to drift. In fact, for most SNPs (65%), their minor allele frequency in the starting population is less than or equal to 2/19 ~ 10%.
- Generally, there is a correlation between |AFC| and our original He (see figure below). However, there are some peaks that are picked exclusively by either one or the other approach. This suggests that the approaches are complementary: while He is clearer when there is a single accession allele that sweeps to fixation, |AFC| can pick regions where SNPs are shared across accessions and thus result in weaker He signals (as the reviewer implied).

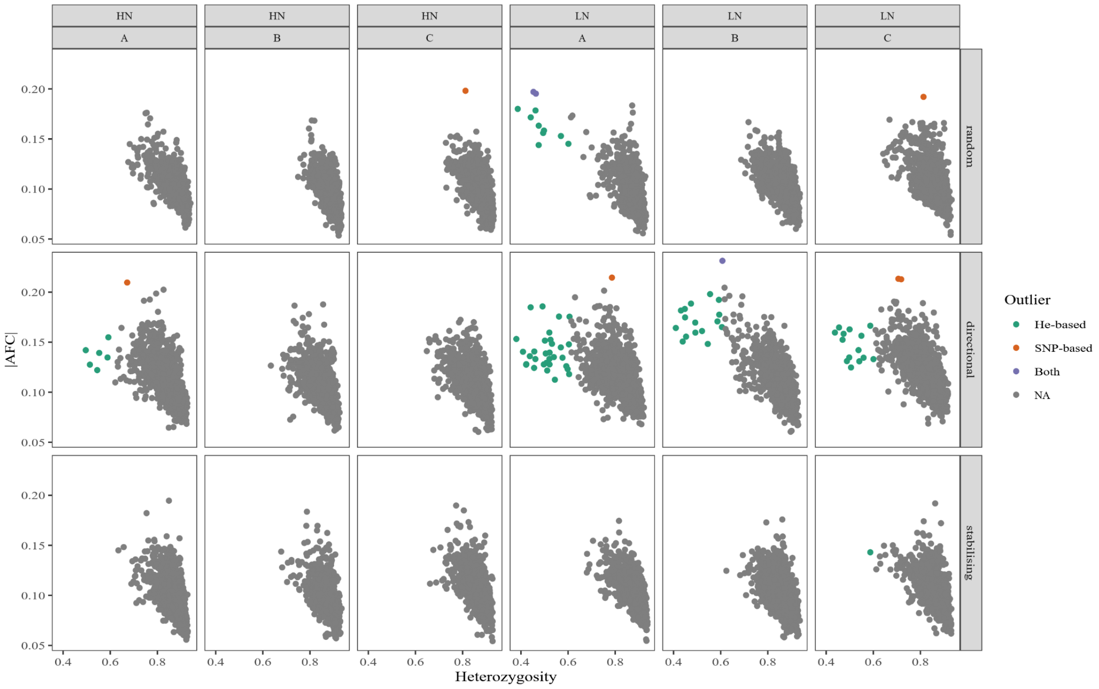


- We expected to have picked these same regions using our modified He statistics (He12, He123, He1234). However, these suffer from the fact that the signal becomes overall noisier (harder to distinguish background from signal), as we said earlier. In that sense, the SNP-based analysis might sometimes pick interesting regions that are missed with our initial He-based metrics.

- We found two new peaks with |AFC| in LN populations. Although we were reluctant to add new figures to what the reviewers already felt was a long paper, we have made a new supplementary figure to include this SNP-based analysis, showing the genome scan of |AFC| as well as the accession frequency distributions in those two new peaks. This is now Fig S10 and we have also made changes to the text accordingly (L466).
- It is noteworthy that using this SNP-based analysis also revealed (presumably) false-positive peaks in random populations. We noticed that this analysis seemed noisier than the He analysis, in particular around the centromeres, where mapping biases are likely more of an issue (we excluded the centromeres when defining outlier peaks).
- In summary, the general conclusions from our genome scans don't change substantially, but we are very grateful for the reviewer's comments, which made us expand and revise our analysis.

Finally, the reviewer suggests looking for consistent changes across population replicates, which we did not observe with any of the approaches taken, as discussed in our response to reviewer #1.

## Reviewer #3

The general aim of the paper is to understand the genetic architecture of shoot branching in Arabidopsis thaliana. The authors have already carried out classical QTL and molecular studies on the trait and they here complement those by carrying out artificial selection experiments. More precisely they created populations by crossing inbred lines (MAGIC lines) and applied different selection schemes: directional selection and stabilising selection over 10 generations under both Low and High nitrate as well as a control population w/o artificial selection. The study included three replicates. They also sequenced the lines at the start and after 10 generations of selection (G10) allowing them to look at signature of selection at the genome level. They focused on selective sweeps although those , given the type of trait studied, do not appear very likely from the start. Overall the paper is a really nice piece of (large!) work, is well written and the analyses were carried out carefully. Perhaps the paper contains too many angles for its own good (e.g the part on the max2 mutant) but I would be inclined to keep them as they also make the paper more complete.

We thank the reviewer for their positive comments on our work. Based on the comments from other reviewers, we have tried to simplify the focus of the paper, which will hopefully address the "many angles" issue mentioned. Namely, we have: 1) shortened the results section by moving incidental discoveries to supplementary appendices and 2) shortened and focused the discussion around the main points on the response to selection in the two nitrate treatments.
Please see our reply to the last comment from the editor, for more details on these changes.

The results are not very surprising: response to directional selection differs under different environments, and shoot branching is likely highly polygenic and redundant (many combinations of loci can give the same result), something that has been observed repeatedly in recent human genetic studies, in particular in a series of recent studies by Jonathan Pritchard's group (e.g. Sinnott-Armstrong, N., Naqvi, S., Rivas, M. & Pritchard, J. K. GWAS of three molecular traits highlights core genes and pathways alongside a highly polygenic background. Elife 10, e58615 (2021) and reference therein). The omnigenic model proposed by Pritchard could be relevant here, at least for discussing the results.

Thanks for this suggestion. This does open interesting research avenues to explore how our candidate loci fit in gene co-expression networks inferred from transcriptome data in shoot branching mutants as well as broader co-expression networks inferred from public data. We have added a new paragraph to our discussion to frame our results in the context of gene regulatory networks of shoot branching architecture (L693).

In general the authors could discuss a bit more the strengths and limitations of their study. For example, one general issue of this type of study that the authors might also want to discuss is the difficulty to draw evolutionary inferences from the type of study they have carried out. In a natural population the distribution of allele frequencies is likely highly different from that in the artificial population they created by crossing MAGIC lines. Since the effect of an allele substitution depends on allele frequencies, there is a risk that the genetic architecture detected in artificial populations and under artificial environmental conditions only has a vague resemblance to that observed under natural conditions. This departure between artificial and natural site frequency spectra can, for instance, explain why epistatic variance matters in population created by crossing lines fixed for alternative alleles while it does not seem to be significant in other populations (Hill, W. G., Goddard, M. E. & Visscher, P. M. Data and theory point to mainly additive genetic variance for complex traits. Plos Genetics 4, e1000008 (2008)). As the authors rightly point out in the introduction "In addition, allelic diversity at each locus, population structure, and the effect of environment on the traits under study all complicate the analysis" but it also strongly impact the meaning of the conclusion that can be drawn. So the authors could increase a bit the scope of the discussion and in particular place their results in a broader context.

Again, many thanks for this suggestion, which was also mentioned by reviewer #4. We have included a new paragraph in our discussion to discuss these issues (L667):

“The question remains about whether the trait complexity described here is of relevance in wild populations, as that would require, for example, performing field experiments using ecotypes adapted to different environments, assessing the frequencies of relevant alleles in those populations and where they might be adaptive (Lopez-Arboleda et al. 2021; Kerwin et al. 2015; Verhoeven et al. 2004). The same is true for the issue of epistasis just mentioned: while this may be of relevance in breeding experiments, it may bear no significance in the evolution of wild populations, in particular if the allele frequencies at the interacting loci make it unlikely for an individual to carry the right combination of alleles (Hill et al. 2008).”

Minor comments:

Figure S1 is very helpful and could be integrated in the main text.

We have now included this as Fig 1A (and relabelled the remaining panels accordingly).

Line 422. This will not be correct if there is balancing selection and it should be pointed out that there will be a large variation among loci/haplotypes due to drift.

We have adjusted the text to now say (L296, addition in bold): “This measure of genetic diversity should be lower at a selected locus compared to the rest of the genome, **assuming the selected allele rises to (near) fixation**.”
We have also discussed further issues of statistical power in our scans, as highlighted in the reply to other reviewers.

## Reviewer #4

Tavares & Readshaw et al. investigated the genetic basis of branch number in Arabidopsis. The authors’ goal was to understand the genetic architecture of shoot architecture and plasticity in shoot architecture through a very ambitious select and sequence strategy. To accomplish this, the authors used an Arabidopsis MAGIC population with 19 founders. They then selected this population for 10 generations under nitrogen limited and high levels of nitrogen. They conducted both stabilizing and directional selection. As expected, response to selection was only seen in the directional selection populations. Sequencing the populations at the end of selection allowed the authors to establish the genetic architecture that was important for changes in shoot architecture under each selection regime. Overall, there were 18 populations that were selected upon, which were broken into sets of three replicates for each type of selection regime. The authors found that a different genetic architecture was under selection in low nitrogen selections in contrast with the high nitrogen selections. Low nitrogen suppresses branching. From genome-wide data, the authors identified genomic regions that were likely under selection over the generations of the project. These results contrasted between selection in high and low nitrogen. To better understand those results, the authors conducted computational simulations. Finally, the authors discovered a new mutation in one of their experimental populations that affected shoot architecture. Using complementation studies, they showed that the new mutation was in the MAX2 gene.

I am overall very impressed with the study design and the amount of work that went into crosses, data collection, and analysis. The manuscript involves a thoughtful analysis of how different the genetic architecture under selection was depending on type of selection and nutrient content.

We thank the reviewer for their positive view of our work.

While the manuscript is generally easy to read, it is quite long and I found myself struggling to maintain comprehension of all the sections of the results as I read the manuscript. I wonder if it would be worthwhile to try to reduce and combine some of the sections of the results to make the whole of this large body of work more digestible for the average reader. That said, I do think all of the content for this manuscript should be published as a single unit, as it would also be difficult to understand each of the pieces of the study in isolation.

This was a comment common to all reviewers as well as the invited editor. As mentioned in the reply to the other reviewers, we have attempted to streamline the focus of the manuscript, which we hope helps address this issue.
Please see our reply to the last comment from the editor, for more details on these changes.

Additional comments:

There were a number of minor typos (e.g. extra periods at the ends of sentences) throughout the manuscript. Make sure to correct them with revisions.

We have fixed as many of these as we could find and will fix any further typos during the final editing stages.

22: “where previous association mapping failed to recover much of its heritability” is confusing; perhaps “where loci identified by association mapping failed to explain heritability estimates”

Thank you for this suggestion, we have amended accordingly.

188: If median absolute deviation is equivalent to standard deviation, why not use standard deviation?
188, 259 407: it may be better to say “analogous” or “similar” instead of “equivalent” here, since standard deviation and median absolute deviation will give different values

Thanks for pointing this out. We agree that “analogous” is a better word and have corrected it throughout.

261-262: I am a bit confused as to why drift is being equated with random selection here rather than being defined as a sampling error based on population size

Thank you for this observation. We have amended our text to mention random selection only, which is what we meant (without conflating this with drift effects, which may be at play in any population, regardless of their selection regime).

Fig. S6: The threshold here looks the same as the threshold used for directionally selected populations, yet stabilizing selection resulted in higher than expected heterozygosity. Shouldn’t this figure use a different threshold calculated from populations under stabilizing selection? A few peaks (chr 1 LN B and C, chr 4 HN A and B) look like they may be close, and an overly stringent threshold could falsely indicate that there was no detectable effect of selection (line 487) on heterozygosity in populations under stabilizing selection.

We do indeed use the same threshold for all populations. The reasoning is explained in the methods section “Thresholds for identifying candidate selective sweeps”. Briefly, we use the threshold defined from populations under directional selection to avoid a high rate of false positives (based on control populations). As we have found no evidence that stabilising selection had an impact on those populations (based on inbreeding coefficients, and analysis of trait variances), we chose not to relax our thresholds in the scans for those populations. However, as pointed out, we may have been overly conservative.
When defining thresholds based on simulation (Fig S7C) those loci do indeed show as outliers. We have therefore updated Fig S7C to include the stabilising selection scans (the

previous version omitted these) and updated the text accordingly (L427). This figure has also been improved by highlighting the regions that fall below the threshold with an arrowhead (this addresses a minor point raised below).
However, we have opted to keep the more conservative threshold as the main figure, for the reason pointed above.

Fig. 6A: right hand side (chromosome numbers?) got cut off

This has been fixed.

516: On first reading, this sentence appears to contradict the one on line 513. Perhaps “not biased towards any one particular accession haplotype among different selective sweeps”

We have clarified this to say (L391): “Selection was not always for the same accession haplotype (...)”

Fig. S7C: What do the black points indicate?

These were the windows that fell below the threshold. We added this since some of the windows are quite close to the threshold, making it harder to see them with just the lines. We have improved this figure to include arrowheads instead and have modified the legend accordingly. As mentioned above, we have also now included the stabilising selection scans to this figure.

Fig. S10Aiii, Biii: It would be easier to compare these two panels if the x-axes covered the same range.

We have made the axis the same across panels. We note that this figure has also been revised, as detailed in our reply to the editor.

723: This is a serendipitous thing that happened and it brings me great joy that the authors decided to explore it.

Many thanks for the positive comment. We note that, in order to streamline the narrative and reduce the main text, we have moved this section to S2 Appendix. However, we kept the same detailed description of the results for any interested readers.

785: It might be good to clarify the statement “However, most of the genetic variation was not recovered.” to make it clear that loci identified in the study only explained a fraction of the genetic variation in the trait.

We now say (L616): “However, only a fraction of the heritability estimated for shoot branching in that study was explained by the QTL identified”

815: I don’t understand this sentence- what is the robustness of branching traits?

Apologies, we were thinking of the relationship between trait means and their variability, in particular in light of previous work focusing specifically on natural variation for seed germination variability. We have now rephrased it as (L563):
“Overall, this suggests a weak genetic basis for shoot branching variability, unlike what has

been described for example in seed germination (Abley et al. 2021; Sharma and Majee 2023).”

823: I was not previously aware of the Jinks-Connolly rule. This is an interesting point. Also, make sure to spell Connolly correctly in the revision.

Thanks, we have fixed this typo.

854: Please clarify what is meant by “axillary meristem formation.” Do you mean initiation of axillary branches from axillary buds?

There are two distinct aspects involved: the initiation of the meristem itself and then the regulation of their dormancy/activation. Our study concerns the regulation of shoot activation, but we wanted to discuss that the initiation of meristems can itself be variable. We have attempted to clarify this, by now saying (L601):
“We note that flowering time has also been implicated in axillary meristem formation, thus the initiation of meristems themselves may be a source of variation in shoot architecture in addition to the regulation of bud dormancy.”

882-885: How much do you think that this being a MAGIC population with such a large number of founder alleles lead to this result of different loci being selected upon in different populations? It seems like the level of standing genetic variation and low allele frequencies at causative loci would not be very representative of “natural” Arabidopsis populations. I realize that mimicking natural populations was not the goal here, but it might be worth writing some reflections on how the MAGIC design itself influenced the results. The authors do do this on line 966-969, but they may want to make it more explicit here as well or instead.

Many thanks for raising this issue, which was also mentioned by reviewer #3. We have now added a new paragraph to our discussion (L667), which says:

“The question remains about whether the trait complexity described here is of relevance in wild populations, as that would require, for example, performing field experiments using ecotypes adapted to different environments, assessing the frequencies of relevant alleles in those populations and where they might be adaptive (Lopez-Arboleda et al. 2021; Kerwin et al. 2015; Verhoeven et al. 2004). The same is true for the issue of epistasis just mentioned: while this may be of relevance in breeding experiments, it may bear no significance in the evolution of wild populations, in particular if the allele frequencies at the interacting loci make it unlikely for an individual to carry the right combination of alleles (Hill et al. 2008).”
