# Supplementary material for: Artificial selection reveals complex genetic architecture of shoot branching and its response to nitrate supply in Arabidopsis
Source: PLoS Genet. 2023 Aug 24;19(8):e1010863. doi: 10.1371/journal.pgen.1010863 (PMC10482290; doi:10.1371/journal.pgen.1010863)
Supplement: Attachment — Submitted filename: 2023-06-17 Response to reviewers.pdf [file pgen.1010863.s019.pdf]

Guest editor

The manuscript is clearly massively improved, and I see no reason not to publish. As noted by Reviewer 2, there are still several things one could quibble about. These, I believe, mostly stem from writing a paper in a different field with a huge literature. That said, the reviewer is right, and reading a few more papers wouldn't hurt. But I leave this up to you. There are many such examples, e.g. the notion of associative overdominance, which you seemingly attribute to a 1998 paper, but which I'm confident is far older (the 1998 paper cites Frydenberg, 1963, but I guess the notion is in Fisher somewhere). Anyway, we're not doing history of science here, and the readers can figure it out.

Indeed, it seems that the concept of “overdominance” was coined by Frydenberg 1963 (cf. Zhao & Charlesworth 2016), based on their study of a lethal allele in the *Stubble* locus in *Drosophila*, which remained at high frequencies (rather than lost) in artificial populations kept in the lab. The maintenance of this polymorphism was later shown to be due to linkage to a nearby inversion, possibly acting as a balancer (although we did not dig further into the history of these specific lines). We have now added Frydenberg as a reference.

One slight thing I would fix is l. 41, "[o]ur results suggest that this trait is controlled by tens of loci of small effect" — you mean "a minimum...". The bottom line is that you have limited power to detect really small effects, and that your resolution is limited by haplotype structure and linkage disequilibrium, which means that your sentence if by "loci" you mean QTL, e.g. large haplotypes with unknown numbers of causative polymorphisms.

Thanks for highlighting this. We have amended this throughout the text, to clarify that these are lower-bound estimates. In addition, we have added (L625):

“Our estimate for the number of QTL is a lower bound, as there is low power to detect very small effects. In addition, multiple causative loci may in fact occur on the same selected haplotype block, and we have no resolution to distinguish this from linked selection on neutral loci (Smith and Haigh 1974; Nuzhdin and Turner 2013).”

Reviewer #1

The authors have addressed my comments and the revised manuscript is easier to follow.

Many thanks.

Reviewer #2

First, I would like to thank the authors for the careful consideration given to my comments and those of the other reviewers. I am convinced by their arguments that there is little signal of parallel change across their selection populations. This supports their heterozygosity analysis as a useful summary of results.

Many thanks for the detailed comments, and we are glad that our revisions improved the manuscript.

The authors argue that genetic redundancy led to a genetically heterogeneous response to selection. Given this conclusion, my only advice is to broaden the discussion of genetic redundancy. In truth, the empirical evidence for genetic redundancy in quantitative traits is NOT "extremely sparse." Redundancy is built directly into most multi-locus genetic models. It is supported by multiple lines of evidence for quantitative traits in many systems (see summaries in Falconer and Mackay or Lynch and Walsh). For a nice example in the specific context of selection experiments, the authors might revisit several papers from F.M. Cohan published in Evolution around 1984 (in D. melanogaster), but there are many other examples.

Many thanks for the additional suggestions. We have added references to those earlier works on selection for ethanol tolerance in Drosophila melanogaster and pseudoobscura, as well as others. Critically, we have added a reference to a very timely review published just a few months ago by Christian Schlötterer on this precise topic.

We have modified our text as follows (L640):
"There are many examples in Drosophila where experimentally imposed pressures result in correlated trait responses across replicates, but with distinct genetic outcomes (reviewed in (Schlötterer 2023)). From earlier studies of ethanol tolerance (Cohan and Hoffmann 1986; Hoffman and Cohan 1987) to more recent studies of desiccation tolerance, adaptation to hot environments, amongst others (Michalak et al. 2019; Barghi et al. 2019; Griffin et al. 2017). While the population of origin is critical in the genetic response to selection (Seabra et al. 2017), even populations with similar founders can show parallel evolution for a trait through selection for different loci. Simulations show that this pattern can be explained by genetic redundancy, whereby populations converge toward similar phenotypes with different underlying allelic combinations across many loci, as long as the founder population has enough genetic diversity and the trait has a relatively complex genetic architecture (Barghi et al. 2019; Barghi and Schlötterer 2020; Burny et al. 2021). This is compatible with our own data and simulations."

We have also added (L661):
"It remains to be clarified how the genotypic redundancy described here relates to the underlying developmental pathways controlling shoot branching, in particular with respect to the interaction with nitrate availability. Is the genotypic redundancy associated with functional redundancy (e.g. loci may be functionally redundant if they affect rate-limiting steps of the same biochemical pathway), or are different pathways involved in regulating this trait (Láruson et al. 2020)? A study of inbred lines of Arabidopsis suggests that functional redundancy is not common in this system (Rutter et al. 2017). However, this study focused primarily on t-DNA lines, which are likely to carry knockout mutations and thus it remains unclear whether natural alleles affecting different developmental programs show functional redundancy in this system. Further dissection of the causal loci affecting shoot branching variation may help address this question in the future."

Reviewer #3

The authors have accounted for my comments and those of the associate editor and of other reviewers and the paper can now be published.

Many thanks.
